# Hydration and Conformation of 2‑Ethylfuran Explored by Microwave Spectroscopy

**DOI:** 10.1021/acs.jpca.5c01281

**Published:** 2025-05-14

**Authors:** Charlotte N. Cummings, Nicholas R. Walker

**Affiliations:** Chemistry- School of Natural and Environmental Sciences, Newcastle University, Bedson Building, Newcastle-upon-Tyne NE1 7RU, U.K.

## Abstract

Rotational spectra of one conformer of a 2-ethylfuran···H_2_O complex and two conformers of the isolated 2-ethylfuran molecule have been recorded by chirped-pulse Fourier transform microwave spectroscopy. The species were probed while entrained within a gas sample undergoing supersonic expansion. The spectra of five isotopologues of the complex have been analyzed to yield rotational (*A*_0_, *B*_0_, *C*_0_) and centrifugal distortion constants (*D*_*J*_, *D*_*JK*_, *d*_1_) allowing structural parameters to be determined by fitting to the experimentally determined moments of inertia. Quantum chemical calculations have been performed to support the interpretation of the experimental results and gain further insights. 2-Ethylfuran is shown to adopt C_1_ symmetry within the observed conformer of 2-ethylfuran···H_2_O with the length of the hydrogen bond, *r*(H_b_···O1), which connects H_2_O with 2-ethylfuran determined to be 2.0950(42) Å in the *r*_0_ geometry. The geometry of the hydrogen bonding interaction deviates from linearity such that the ∠(O_w_–H_b_···O1) angle (where O_w_ and O1 are the oxygen atoms of water and furan, respectively) is 167.69(16)° in the *r*_0_ geometry. The experimental and theoretical results thus imply the presence of a weak interaction between the oxygen of H_2_O and the ethyl group within the observed conformer of 2-ethylfuran···H_2_O. Evidence is presented to suggest that the C_s_ conformer of the isolated 2-ethylfuran molecule is lower in energy than the C_1_ conformer implying that the energy ordering of the two lowest-energy conformers of 2-ethylfuran reverses when the isolated molecule is hydrated by a single H_2_O molecule.

## Graphical abstract

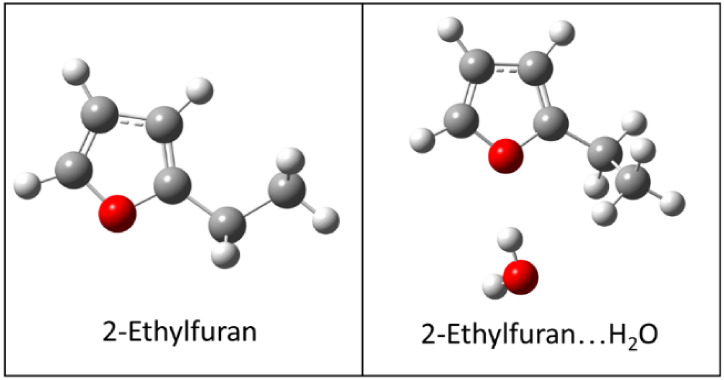

## 1 Introduction

Heteroaromatic rings are a component of many biochemically important species and can participate in hydrogen bonding, π-stacking and other forms of intermolecular interaction. Water is ubiquitous in biochemical systems. It is therefore important to understand the nature of intermolecular interactions between H_2_O and heteroaromatic rings and the extent to which these influence broader aspects of molecular structure. The present work applies microwave spectroscopy to determine the geometry of a 2-ethylfuran···H_2_O complex generated and isolated in the cold environment of a gas sample undergoing supersonic expansion. The aim is to provide detailed insight into the nature of intermolecular interactions between H_2_O and 2-ethylfuran (hereafter denoted as 2-EF) and the consequences for molecular conformation. Observations that further the understanding of conformer stability for the isolated 2-EF molecule will also be described.

Microwave spectroscopy yields precise information about shape, structure and conformation in small and isolated molecular units. For example, studies have explored conformational preferences resulting from competing, noncovalent, intramolecular interactions in molecules where an alkyl or other substituent is present on a heteroaromatic ring.^1–4^ The energy ordering of conformers of such molecules depends on electronic and geometric effects that are mediated by the heteroatom(s). A conformer of ethylbenzene that has been detected experimentally has C_1_ symmetry where the dihedral angle that defines the orientation of the ethyl group relative to the ring (analogous to the ∠(C7–C6–C2–O1) angle shown in Figure 1) is 90°. In contrast, two distinct conformers (respectively having C_s_ and C_1_ symmetry) of 2-EF have been observed where the sample probed was entrained within a helium carrier gas.^3^ Within the C_s_ conformer of 2-EF, the heavy atoms of the ethyl group were found to be coplanar with the furan ring such that the ∠(C7–C6–C2–O1) dihedral angle is equal to 180°. In the C_1_ configuration, ∠(C7–C6–C2–O1) was determined to be 63.31(64)° (in the *r*_s_ geometry). A recent study on 2-ethylthiazole^4^ observed only the C_1_ conformer of this molecule during a series of experiments performed using argon and neon buffer gases with the value of ∠(C7–C6–C2–S1) (with the numbering of atoms within thiazole being analogous to that shown in Figure 1) determined to be 98.6(10)°.

**Figure 1:**
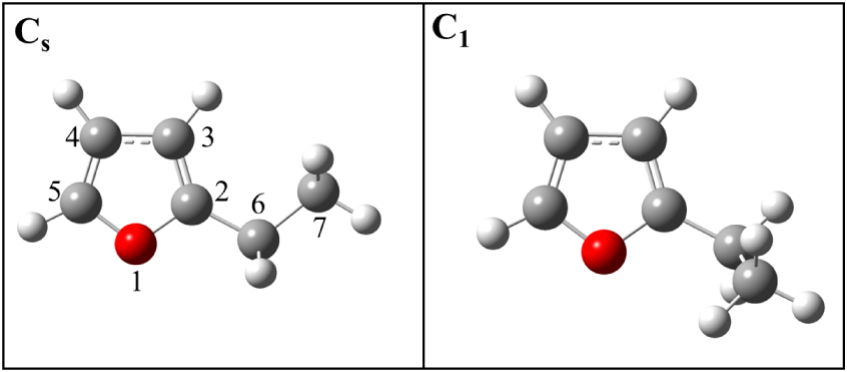
Two observed conformers of 2-ethylfuran reported in reference 3. This work uses numeric labels for C and O atoms as shown (left).

Furfuryl alcohol,^5,6^ furfuryl mercaptan^6,7^ and furfuryl amine^8^ differ from each other and from 2-EF in respect of the substituent attached to C6. Replacing the CH_3_ group of 2-EF by OH, SH or NH_2_ leads to the structures of furfuryl alcohol, furfuryl mercaptan or furfuryl amine, respectively. For each molecule in this series, the lowest-energy geometry possesses an X–H···O1 intramolecular hydrogen bond (where X is O, S or N). The values of ∠(X–C6–C2–O1) within the lowest energy conformers of furfuryl alcohol and furfuryl mercaptan are 66.47(43)° and 71.81(38)° respectively when determined from the *r*_0_ coordinates reported by the original work. The same parameter is 65.37(23)° in furfuryl amine from the partial *r*_s_ geometry.^6,8^ Markedly different results were obtained during experiments performed to study 2-methoxyfuran.^9^ The experimentally observed conformer of 2-methoxyfuran has *C*_*s*_ symmetry with the methoxy group lying within the plane defined by the furan ring (∠(C–O–C2–O1) = 180°). It was suggested that delocalization of π-electrons onto the oxygen atom stabilizes the geometry of this conformer relative to others.

Upon formation of a 1:1 complex between subunits A and B, intermolecular interactions can significantly influence the overall energy of a complex. The lowest-energy conformation of an A···B complex might *not* contain those conformers of A and B that are found to have lowest energy when each of A and B is isolated. Hence, when B is H_2_O, the conformer of A within an A···H_2_O complex might differ from that observed for isolated A. Several studies have examined conformer energy ordering for hydrated complexes^10–16^ while others have identified a shift in tautomeric equilibrium. During the present work, the microwave spectrum of a monohydrate complex of 2-ethylfuran (hereafter denoted as 2-EF···H_2_O) will be presented and analyzed for the first time. It will be shown that the lowest-energy geometry of this complex contains 2-EF in a conformation (C_1_) different from that which is the lowest-energy conformer (C_s_) of the isolated 2-EF molecule. The results will be compared with those of previous works which have studied monohydrate complexes of 2-ethylthiazole,^4^ furfuryl alcohol^6^ and furfuryl mercaptan.^6^

## 2 Methods

### 2.1 Experimental Methods

2-Ethylfuran and a low concentration of H_2_O were initially entrained within either an argon (BOC, 99.998%) or neon (BOC, CP grade) carrier gas. The resulting gas sample underwent supersonic expansion from a pulsed nozzle (Parker, Series 9) and chirped-pulse Fourier transform microwave (CP-FTMW) spectra (spanning 7.0 – 18.5 GHz) were recorded for species present in the expanding sample. 2-EF (Alfa Aesar, 98%) was seeded directly into the flow of a carrier gas (before the supersonic expansion) from a bespoke reservoir.^17^ The vapor pressure of 2-EF (54 mmHg @ 298 K) at ambient temperature is sufficiently high that it was not necessary to heat the reservoir when introducing 2-EF into the gas sample. Water was introduced into the carrier gas flow from a second reservoir (again, it was not necessary to heat the reservoir above room temperature) to encourage the formation of hydrate complexes. Experiments performed using a neon carrier gas and either D_2_O (Sigma-Aldrich, 99.9% D atom) or H_2_^18^O (Sigma-Aldrich, 97% ^18^O atom) allowed measurement of the spectra of D- and H_2_^18^O-containing isotopologues of 2-EF···H_2_O.

The CP-FTMW spectrometer at Newcastle University has been described in detail elsewhere.^18,19^ During operation of the instrument, a chirped pulse of 1 μs in duration (spanning from 0.5 to 12 GHz in frequency) is generated by a 20 GS s^–1^ Arbitrary Waveform Generator (AWG) (Tektronix AWG 7102). The microwave radiation is mixed against a 19 GHz reference signal provided by a Phased Locked Dielectric Resonant Oscillator (PDRO). The lower frequency sideband (7.0–18.5 GHz) is selected by a low pass filter and the higher frequency sideband (19.5–31.0 GHz) is subsequently removed. The microwave radiation is amplified using a 300 W Traveling Wave Tube Amplifier (TWTA) before broadcasting into the vacuum chamber via a horn antenna. The pulse of microwave radiation intersects the expanding gas sample and rotationally polarizes molecules and complexes on resonance with rotational transitions. The free induction decay (FIDs) of the molecular emission is recorded at a second horn antenna over a duration of 20 μs. Eight FIDs per nozzle pulse are digitally recorded by a 100 GS s^–1^ oscilloscope (Tektronix DPO72304XS). All FIDs are coadded together in the time domain before a Fourier transform of the data is performed using a Kaiser-Bessel window function. A line width of 100 kHz is achieved for an isolated line at full width half-maximum with an estimated accuracy of 10 kHz in the line center frequencies in the frequency domain spectrum. Phase coherence in the time domain and accuracy in transition frequencies are provided by an Analog Signal Generator (Agilent MXG N5183A) to which the AWG, the PDRO and the oscilloscope are phase-locked.

### 2.2 Theoretical Methods

Quantum chemical calculations were performed using the Gaussian09 package.^20^ Potential energy scans used the harmonic hybrid function^21–24^ of Becke, Lee, Yang and Parr (B3LYP), in conjunction with Grimme’s dispersion correlation effects^25^ and the Becke-Johnson damping function,^26,27^ D3BJ, alongside either Dunning’s augmented triple-ζ aug-cc-pVTZ basis set,^28,29^ B3LYP­(D3BJ)/aug-cc-pVTZ, or Ahlrichs^30,31^ valence polarized Def2-TZVP basis set, B3LYP­(D3BJ)/Def2-TZVP. A scan calculation used second order Møller–Plesset perturbation theory^32^ with Dunning’s augmented double-ζ aug-cc-pVDZ basis set, MP2/aug-cc-pVDZ. Additional calculations employed MP2/6-311++G­(d,p) and MP2/6-31G­(d,p) levels of theory which were used during a previous study on 2-EF.^3^ Each scan explored the variation of the potential energy of the 2-EF monomer with the ∠(C7–C6–C2–O1) dihedral angle through potential energy calculations performed at 5° intervals. Each calculation predicts three minima (as shown in Figure 2) of which two are mutually equivalent by symmetry. The minima thus represent the C_s_ and C_1_ conformers identified in the 2020 study.^3^ The geometry of each conformer was initially optimized at the B3LYP­(D3BJ)/aug-cc-pVTZ level and subsequently reoptimized using the long-range corrected hybrid functional, ωB97X-D, with Dunning’s quadrupole-ζ aug-cc-pVQZ basis set (ωB97X-D/aug-cc-pVQZ).^33^ Rotational constants, dipole moment components and atomic coordinates calculated at each level of theory are provided in Tables S1–S5.

**Figure 2:**
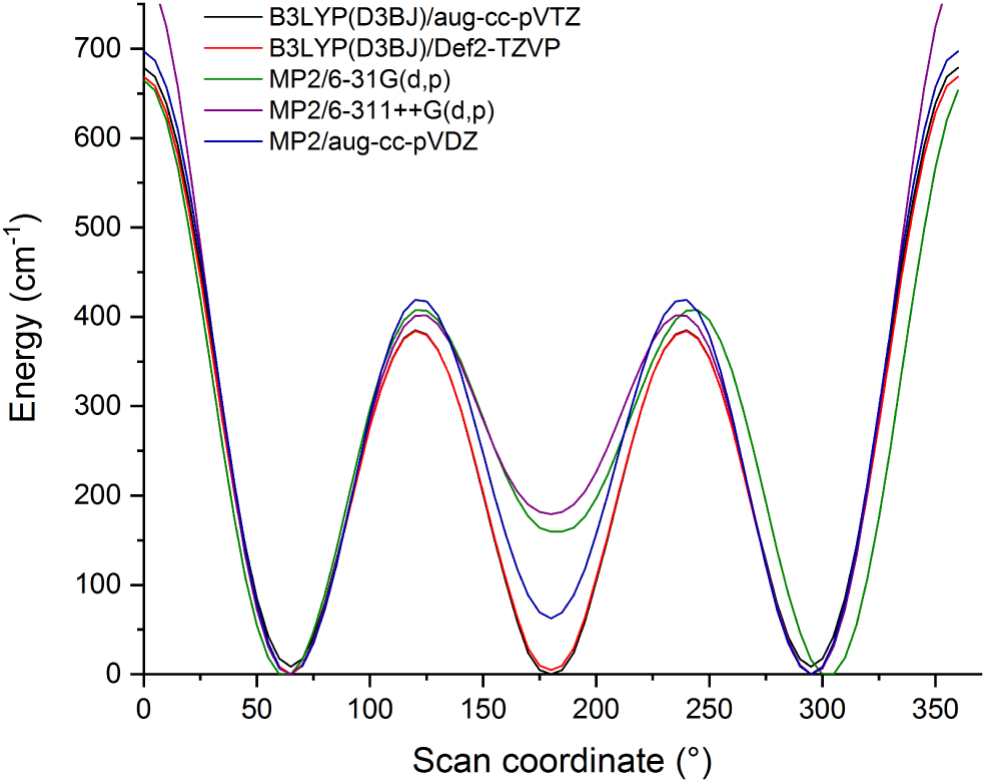
Potential energy scans obtained by rotating the ethyl group relative to the furan ring in 2-ethylfuran. Calculations performed at the B3LYP­(D3BJ)/aug-cc-pVTZ, B3LYP­(D3BJ)/Def2-TZVP, MP2/6-311++G­(d,p), MP2/6-31G­(d,p) and MP2/aug-cc-pVDZ levels of theory by scanning the ∠(C7–C6–C2–O1) dihedral angle (energies calculated at 5° intervals).

Each geometry optimization started by placing the H_2_O molecule close to the oxygen atom of 2-EF with the geometry of the latter initially chosen to correspond to an identified minimum on the potential energy surface of 2-EF. At the B3LYP­(D3BJ)/aug-cc-pVTZ level, the C_s_ conformer (as denoted in Figure 3) was calculated to be 1.01 kJ mol^–1^ higher in energy than the C_1_ conformer. Additional geometry optimizations were performed at the ωB97X-D/aug-cc-pVQZ, B3LYP­(D3BJ)/Def2-TZVP and MP2/aug-cc-pVDZ levels. Rotational constants (*A*_e_, *B*_e_ and *C*_e_) and dipole moment components (μ_a_, μ_b_, μ_c_) are given in the Tables S6 and S7, for each conformer. Atomic coordinates calculated at each level of theory are provided in Tables S8–S15.

**Figure 3:**
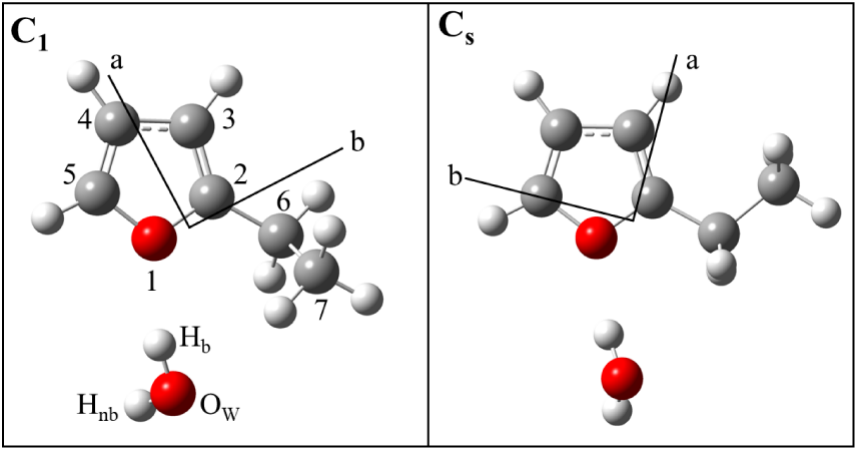
Equilibrium (*r*_e_) geometries of hydrate complexes formed with the C_1_ (left) and C_s_ (right) conformers of 2-EF as calculated at the ωB97X-D/aug-cc-pVQZ level of theory.

## 3 Results

### 3.1 Spectral Assignment and Analysis

Figure 4 displays microwave spectra recorded between 14665 and 15250 MHz. A spectrum recorded while using neon (Figure 4, top) contains *a*- and *b*-type transitions of C_s_ and C_1_ conformers of the 2-EF monomer. The observation of these conformers under these experimental conditions is consistent with a previous work which also observed both conformers but used helium backing gas.^3^ A spectrum recorded while using argon contains intense transitions of the C_s_ conformer but transitions of the C_1_ conformer are either absent or very weak (Figure 4, bottom). The predominance of the C_s_ conformer in the data recorded when using argon has implications for the energy ordering of conformers which will be discussed further in Section 4. Transitions in the spectra of ^13^C-containing isotopologues of both conformers were observed in natural abundance. Transitions of the ^18^O-containing isotopologue were not observable because of the low natural abundance of ^18^O (0.2%) relative to ^16^O. A significant number of transitions remain in the spectra even after those assigned to conformers of the isolated 2-EF molecule have been removed. It will be shown that many of these transitions assign to a monohydrate complex of 2-EF. Attempts were made to assign the spectra of other hydrate complexes (including polyhydrates) formed with 2-EF but these were unsuccessful.

**Figure 4:**
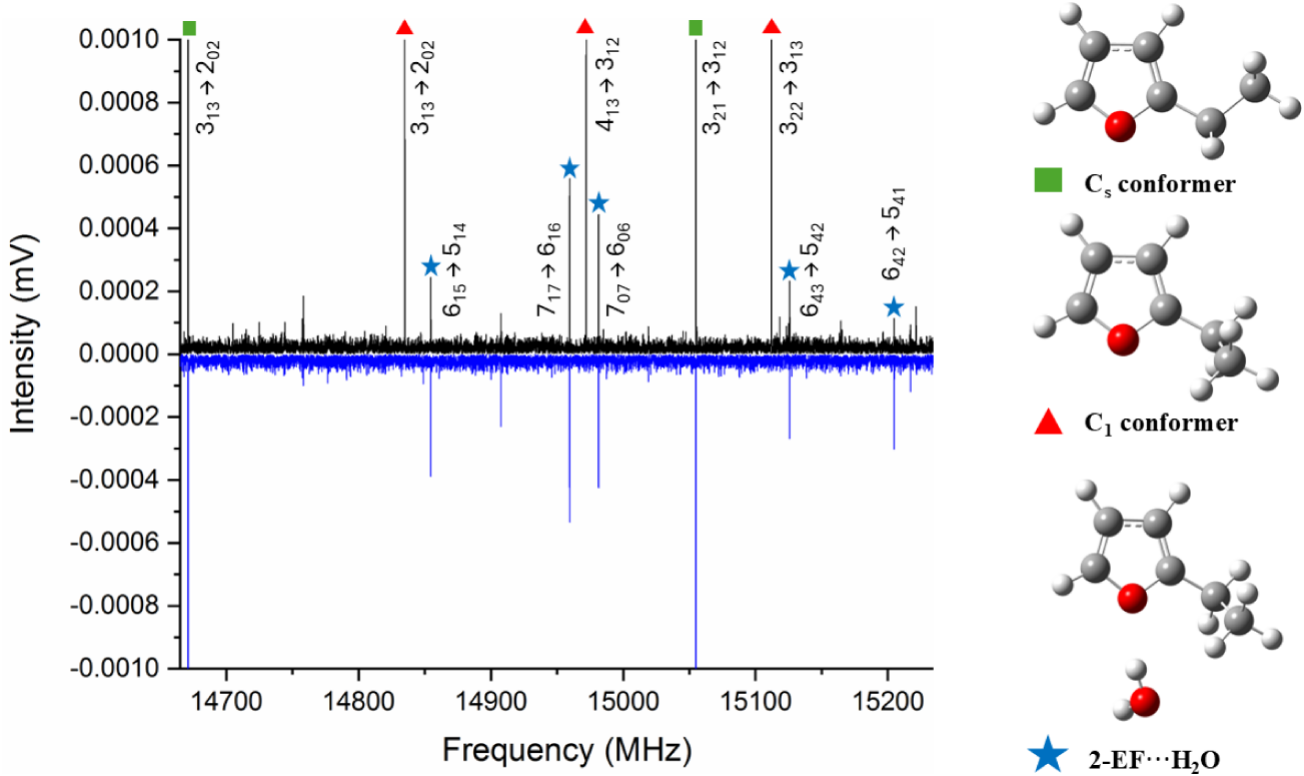
Section of the broadband microwave spectrum covering 14 665–15 250 MHz recorded during experiments that used different carrier gases. The upward trace (black) was recorded while using a neon carrier gas, the downward trace (blue) was recorded while using argon. Transitions of the C_s_ conformer of 2-ethylfuran (green, square), C_1_ conformer of 2-ethylfuran (red, triangle) and 2-EF···H_2_O (blue, star) are assigned with quantum numbers as shown.

Previous studies of complexes formed between heteroaromatic rings and H_2_O have identified many examples of isolated, gas phase complexes where H_2_O acts as hydrogen bond donor and binds to the most electronegative heteroatom (N or O) of the ring. For example, this binding mode was observed for complexes of pyridine,^34^ imidazole,^35^ methylthiazoles,^36^ methylimidazoles,^37^ furan^38^ and furan derivatives^6,39–42^ with water and other small molecules. Quantum chemical calculations identified the C_1_ and C_s_ conformers of 2-EF···H_2_O shown in Figure 3 as having similar energy. Each of these conformers is a near-prolate asymmetric top where *μ*_*a*_ is significantly higher than both *μ*_*b*_ and *μ*_*c*_. Rotational transition frequencies for each conformer were predicted by the results of the quantum chemical calculations. Some transitions were initially observed during an experiment that used argon carrier gas and then seen again during an experiment that used neon. Watson’s *S*-reduced Hamiltonian^43^ as implemented within PGOPHER^44^ was employed to fit the values of spectroscopic parameters on the basis of an assignment of measured transition frequencies. The spectroscopic parameters (including values for the centrifugal distortion constants, *D*_*J*_, *D*_*JK*_ and *d*_1_) determined by fitting to experimentally observed transition frequencies are displayed in Table 1. At the ωB97X-D/aug-cc-pVQZ level of theory, the rotational constants (*A*_e_, *B*_e_, *C*_e_) of the C_1_ conformer were calculated to be 2520.742 MHz, 1483.172 and 1027.281 MHz respectively which are very similar to the experimentally determined results for *A*_0_, *B*_0_ and *C*_0_. Rotational constants of the C_s_ conformer were calculated to be 1926.004 MHz, 1703.675 and 922.305 MHz, respectively. Consistent with expectations for the C_1_ conformer, assigned transitions are *R*-branch (*J* = 3–8) and *a*-type with very few *b*- and *c*-type transitions observed at the sensitivity of the experiments. The barrier to internal rotation of the methyl group of an ethyl substituent^45,46^ is typically found to be greater than 1000 cm^–1^ and the *V*_3_ barrier of the methyl group was calculated to be 946 cm^–1^ at the B3LYP­(D3BJ)/aug-cc-pVTZ level for the C_1_ conformer of 2-EF···H_2_O. It is therefore unsurprising that splittings associated with internal rotation were not observed.

**Table 1:** Experimentally Determined Spectroscopic Parameters for Five Isotopologues of 2-EF···H_2_O

2-EF···H_2_O					
	H_2_^16^O	H_2_^18^O	DOH	HOD	D_2_O
*A*_0_ (MHz)	2530.5118(32)^a^	2473.8961(56)	2502.7183(79)	2470.1215(98)	2445.5277(65)
*B*_0_ (MHz)	1451.9822(11)	1395.45246(56)	1443.35048(91)	1416.6218(10)	1408.16994(72)
*C*_0_ (MHz)	1010.60117(71)	974.62308(63)	1001.07474(90)	984.5466(11)	975.76183(83)
*D*_*J*_ (kHz)	0.673(11)	0.6394(56)	0.6381(85)	0.641(13)	0.6218(76)
*D*_*JK*_ (kHz)	–0.933(53)	–0.514(41)	–0.32(11)	–0.74(13)	–0.630(76)
*d*_1_ (kHz)	–0.1334(68)	[−0.1334]^b^	[−0.1334]	[−0.1334]	[−0.1334]
σ*_rms_* (kHz)^c^	10.2	7.9	10.9	10.9	9.7
*N* ^d^	32	31	26	20	28
*P*_*cc*_ (u Å^2^)	23.84899(25)	23.95407(29)	23.6193(4)	24.0174(5)	23.8061(4)

a Numbers in parentheses are one standard deviation in units of the last significant figure.

b Values in square brackets held fixed at the result for the parent isotopologue, 2-EF···H_2_^16^O.

c Root mean square (rms) deviation of the fit.

d Number of rotational transitions included in the fit.

The recorded spectrum was carefully analyzed for evidence of other conformers of 2-EF···H_2_O but none was found. Confirmation that the observed spectrum should be assigned to the C_1_ conformer of 2-EF···H_2_O was achieved through experiments performed using isotopically enriched samples of D_2_O and H_2_^18^O. Spectroscopic constants determined for 2-EF···H_2_^18^O, 2-EF···DOH, 2-EF···HOD and 2-EF···D_2_O are displayed in Table 1. For each isotopologue, the centrifugal distortion constants *D*_*J*_ and *D*_*JK*_ were determined by fitting while the value of *d*_1_ was held fixed to the value determined previously for the parent isotopologue (2-EF···H_2_^16^O). Observed transition frequencies for each isotopologue are provided in Tables S16–S20.

### 3.2 Molecular Geometry

Even before detailed analysis of the molecular geometry of the observed conformer of 2-EF···H_2_O, significant insights can be gained from planar moments which can be simply calculated. The planar moment, $P_{cc}=\sum m_{i}c_{i}^{2}$ (where *m*_*i*_ is the mass of the *i* ^th^ atom and *c*_*i*_ is its distance from the *ab* plane), is zero or very small if all atoms are located within or close to the *ab* plane of a complex. For the C_1_ and C_s_ conformers of 2-EF···H_2_O featured in Figure 3, *P*_cc_ are calculated to be 24.6 and 5.5 u Å^2^ respectively from the moments of inertia calculated at the ωB97X-D/aug-cc-pVQZ level of theory. The large difference between these values exists because the carbon atom of the methyl group is outside the *ab* plane within the C_1_ but not within the C_s_ conformer. The *P*_cc_ value obtained from the experimentally determined rotational constants of the observed conformer of 2-EF···H_2_^16^O is 23.84899(25) u Å^2^ which supports the proposal that the C_1_ conformer is the carrier of the observed spectrum.

Coordinates (*r*_s_) of the two hydrogen atoms (H_b_ and H_nb_) and oxygen atom (O_w_) of the H_2_O subunit (atom labels as given in Figure 3) can be calculated from shifts in experimentally determined rotational constants on isotopic substitution. Kraitchman’s equations^47^ as implemented within the program KRA are employed to determine such *r*_s_ coordinates.^48^ The results and Costain errors are presented in Table 2 for the C_1_ conformer alongside ωB97X-D/aug-cc-pVQZ-calculated *r*_e_ coordinates. While *r*_s_ parameters are determined from experimental data for the molecule in its ground vibrational state, *r*_e_ parameters are calculated for the equilibrium geometry. Differences between *r*_s_ and *r*_e_ parameter values will (at least in part) depend on the effects of zero-point vibrational motions within the complex. While such effects can be addressed and partially corrected by quantum chemical calculations of anharmonic vibrational frequencies, such calculations lie outside the scope of the present work. The level of agreement between the *r*_s_ and *r*_e_ results herein confirms that the C_1_ conformer is the carrier of the observed spectrum of 2-EF···H_2_O and is similar to that found previously for similar complexes.^35–37,49^ The imaginary value for the (*r*_s_) *c*-coordinate of H_b_ implies that this atom lies very close to the *ab-*plane of the complex. The *c*-coordinate of O_w_ is displaced from the *ab* plane of the complex by 0.2397(63) Å which is consistent with the C_1_ (and not the C_s_) conformer being the carrier of the observed spectrum. The significant differences between the *r*_s_ and *r*_e_ coordinates of the H_nb_ atom arise because zero-point vibrational motions have a greater effect on the coordinates of this atom than on other atoms.

**Table 2:** Comparison of Experimentally Determined (*r*_s_) and *r*_e_ Atomic Coordinates of H_2_O in 2-EF···H_2_O

	Method	*a*/Å	*b*/Å	*c*/Å
H_b_	*r*_e_ (calc.)^a^	1.637693	1.472884	0.050482
	*r*_s_ (exp.)	1.5102(10)^b^	1.57981(96)	[0]^c^
O_W_	*r*_e_ (calc.)	2.571163	1.622233	0.196075
	*r*_s_ (exp.)	2.62469(57)	1.57458(96)	0.2397(63)
H_nb_	*r*_e_ (calc.)	2.660380	2.428093	0.711543
	*r*_s_ (exp.)	2.87490(53)	2.23429(68)	0.4231(36)

a *r*_*e*_ geometry of the C_1_ conformer calculated at the ωB97X-D/aug-cc-pVQZ level of theory.

b Numbers in parentheses after *r*_*s*_ results are Costain errors calculated as δa = 0.015/|a|.

c Imaginary *r*_s_ coordinate obtained, *r*_s_(H_b_) = 0.48559*i, therefore, value of coordinate assumed equal to zero.

Another approach used to determine geometrical parameters (termed *r*_0_ parameters^50^) involves the fitting of bond lengths and angles to the rotational constants determined experimentally for the ground vibrational state of the molecule. The STRFIT^48^ program was used for this purpose during the present work. Again, differences between *r*_0_ and *r*_e_ parameters are expected because the former neglects the effects of zero-point vibrational motions. The fitted parameters were defined exclusively with reference to heavy (C, N, O) atoms to mitigate the uncertainties arising from zero-point vibrations which most significantly affect hydrogen atom positions. The *r*_0_ parameters displayed in Table 3 (and Table S21) were thus obtained while fixing all other geometrical parameters to ωB97X-D/aug-cc-pVQZ (*r*_e_) results. The EVAL program was used to derive the values of internal (*r*_0_) parameters where these could not be explicitly fitted. The interatomic distance, *r*(O_w_···O1), was determined to be 3.0410(34) Å by the *r*_0_ method which compares with the *r*_e_ calculated result of 2.960 Å. The ∠(O_w_···O1–C2) angle was determined to be 111.50(16)° in the *r*_0_ geometry which is similar to the *r*_e_ calculated result of 112.2°. The hydrogen bond distance, *r*(H_b_···O1) and the angles ∠(H_b_···O1–C2) and ∠(O_w_–H_b_···O1) were derived to be 2.0950(42) Å, 115.30(11)° and 167.69(16)° respectively by the described approach. The value obtained for the ∠(O_w_–H_b_···O1) angle implies nonlinearity of the primary hydrogen bond similar to that observed for other complexes formed between heteroaromatic rings and H_2_O. Recent studies of imidazole···H_2_O,^35^ *N*-methylimidazole···H_2_O,^37^ 2-methylimidazole···H_2_O,^37^ 4-methylthiazole···H_2_O,^36^ 5-methylthiazole···H_2_O,^36^ 2-ethylthiazole···H_2_O^4^ each found a ωB97X-D/aug-cc-pVQZ calculated *r*_e_ result for the length of the primary hydrogen bond that is slightly shorter than the experimentally determined *r*_0_ value (typically by 0.02 to 0.05 Å). The difference between the experimentally derived (*r*_0_) and ωB97X-D/aug-cc-pVQZ-calculated (*r*_e_) value of *r*(H_b_···O1) for 2-EF···H_2_O is 0.08 Å which is greater than implied by the range above. It should be noted, however, that the experiments of the present work did not reveal information about the ∠(C7–C6–C2–O1) dihedral angle within 2-EF after formation of the monohydrate. If changes in this parameter are induced by formation of the monohydrate, and particularly if this dihedral is significantly affected by zero-point vibrational motion, this will contribute to uncertainties and the difference between the *r*_0_ and *r*_e_ results for 2EF···H_2_O.

**Table 3:** Comparison of DFT Calculated (*r*_e_) and Experimentally Determined (*r*_0_) Structural Parameters of 2-EF···H_2_O

Parameter	Method	Value
*r*(O_w_···O1)/Å	*r*_e_ (calc.)^a^	2.960
	*r*_0_ (exp.)	3.0410(34)^b^
∠(O_w_···O1–C2) /°	*r*_e_ (calc.)	112.2
	*r*_0_ (exp.)	111.50(16)
*r*(H_b_···O1)/Å	*r*_e_ (calc.)	2.014
	*r*_0_ (exp., derived)^c^	2.0950(42)
∠(H_b_···O1–C2) /°	*r*_e_ (calc.)	116.1
	*r*_0_ (exp., derived)	115.30(11)
∠(O_w_–H_b_···O1) /°	*r*_e_ (calc.)	167.5
	*r*_0_ (exp., derived)	167.69(16)

a *r*_*e*_ geometry calculated for the C_1_ conformer at the ωB97X-D/aug-cc-pVQZ level of theory.

b Numbers in parentheses are one standard deviation in units of the last significant figure.

c Parameters derived using EVAL from *r*_0_ coordinates obtained.

### 3.3 Noncovalent Interactions

The analysis of the experimental data confirms that H_2_O binds to furan at the O1 position and that the observed conformer of 2-EF···H_2_O has a C_1_ geometry in which H_2_O acts as hydrogen bond donor while 2-EF acts as hydrogen bond acceptor. The presence of a nonlinear hydrogen bond implies that additional hydrogen bonding interactions are present within this complex as observed previously for methylthiazole···H_2_O and methylimidazole···H_2_O complexes.^36,37^ Non-Covalent Interactions (NCI)^51^ and Natural Bond Orbital (NBO)^52^ analyses have been performed to visualize the intermolecular interactions present. These were each performed using the optimized geometry of the C_1_ conformer of 2-EF···H_2_O calculated at the ωB97X-D/aug-cc-pVQZ level of theory. Figure 5 displays the NCI plot of the reduced density gradient (RDG) against the sign of the second eigenvalue of the Hessian matrix (λ_2_) of the electronic density (ρ), (sign­(λ_2_)­ρ). A relatively strong, attractive, hydrogen bonding interaction is found between H_b_ of the H_2_O subunit and O1 of the furan ring. There is a larger and more diffuse isosurface between the O_w_ atom of the H_2_O molecule and the ethyl substituent which has areas of weakly attractive and weakly repulsive interaction. The areas of the isosurface indicating weakly attractive interactions are located between O_w_ and the nearest H atoms located on C6 and C7. Evidently, the 2-ethylfuran and H_2_O subunits each act both as hydrogen bond donor and acceptor simultaneously within the geometry of the complex.

**Figure 5:**
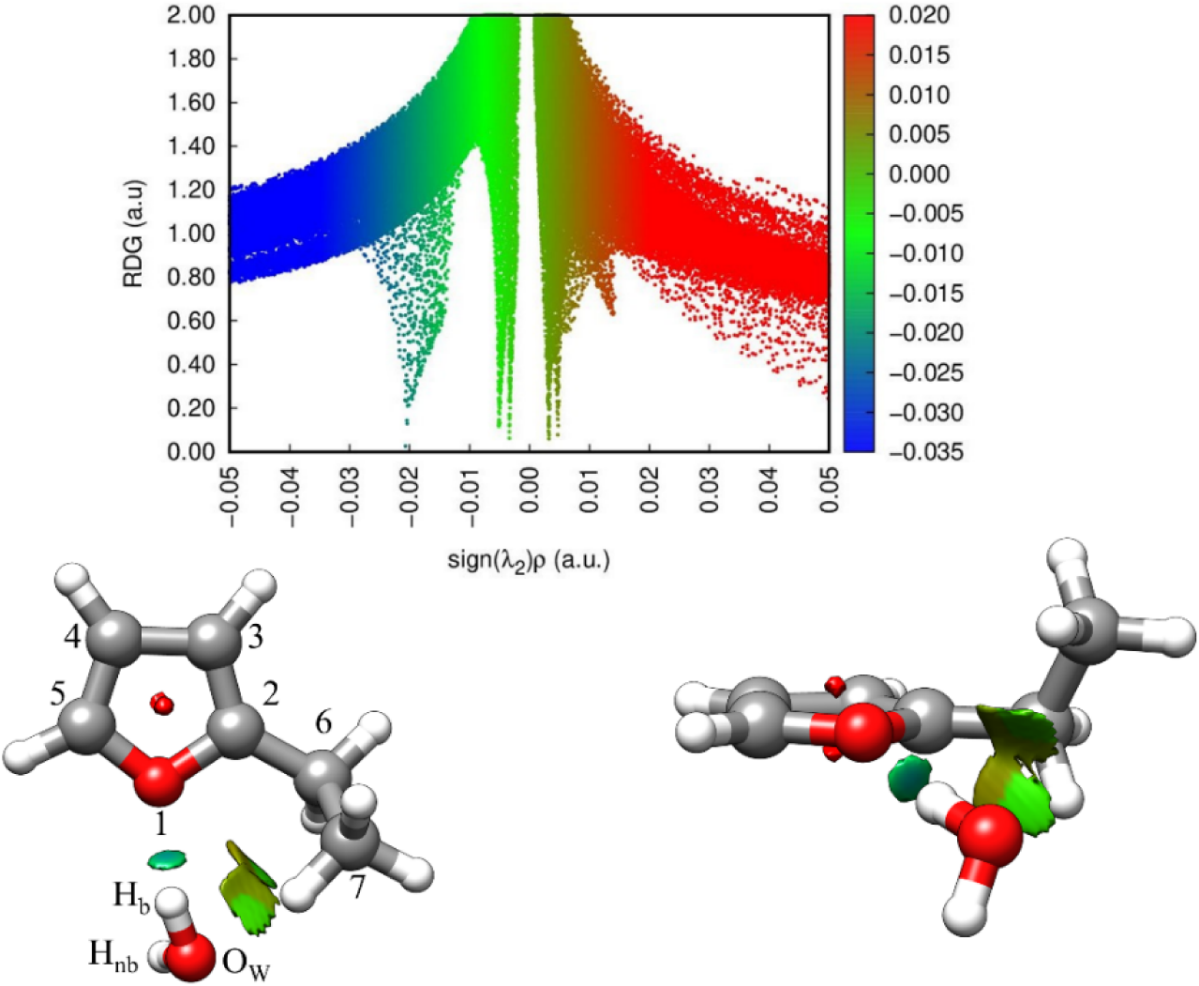
Plot of the RDG (a.u.) vs sign­(λ_2_)­ρ (top) and the NCI isosurfaces (bottom) of the experimentally observed (C_1_) conformer of 2-EF···H_2_O. Positive and negative values of sign­(λ_2_)­ρ respectively denote repulsive (red) and attractive (blue) interactions. The isosurface *s* value is 0.5 au.

NBO analysis was performed at the B3LYP­(D3BJ)/aug-cc-pVTZ level of theory to calculate second order stabilization energies (*E* ^(2)^) (Table S22). The largest *E* ^(2)^ contribution corresponds to the primary hydrogen bonding interaction between one of the lone pairs of the O1 atom and the antibonding σ*­(H_b_–O) orbital of the H_2_O molecule. This interaction was calculated to have a second order stabilization energy of 11.92 kJ mol^–1^. An interaction between the second lone pair on O1 and the antibonding σ*­(H_b_–O) orbital of H_2_O was calculated to be considerably weaker (0.96 kJ mol^–1^). A previous study calculated that the primary hydrogen bonding interaction within furan···H_2_O has *E* ^(2)^ contribution of 30.42 kJ mol^–1^ (when calculated at the MP2/6-31+G* level of theory).^53^ When the structure was reoptimized at the ωB97X-D/aug-cc-pVQZ level of theory and NBO analysis subsequently performed (at the B3LYP­(D3BJ)/aug-cc-pVTZ level), the *E* ^(2)^ of the primary hydrogen bond was calculated to be 8.5 kJ mol^–1^ which is closer to the value obtained during this work for 2-EF···H_2_O.

The NBO analysis finds that each lone pair on O_w_ forms an attractive interaction with a hydrogen atom on C6. The second order stabilization energies of the resultant interactions are calculated to be 0.88 and 0.33 kJ mol^–1^. The weakly attractive interaction between O_w_ and the nearest H7 atom, which was suggested by the NCI analysis, was not confirmed by the NBO analysis (when performed for the ωB97X-D/aug-cc-pVQZ optimized geometry). This inconsistency between the NCI and NBO analysis likely arises due to the interaction being very weak. This was also observed for thiazole···H_2_O^49^ and 5-methylthiazole···H_2_O^36^ where the determined values of the ∠(O_w_–H_b_···O1) angle implied that a secondary, weaker hydrogen bonding interaction (C2–H2···O) was present even though not detected by either NCI or NBO analysis.

NCI and NBO analyses were performed for the C_s_ conformer of 2-EF···H_2_O to explore why this conformer has higher energy than the C_1_ conformer. The NCI analysis (see Figure S1) revealed the presence of three isosurfaces in the C_s_ conformer. Alongside the primary hydrogen bonding interaction (between O1 of the furan ring and H_b_ of H_2_O), there is a weakly attractive interaction between O_w_ of H_2_O and an H atom on C6. It is not possible for the H_2_O molecule to interact with the H atoms attached to C7 given that the heavy atoms of the ethyl group are in the *ab* plane and oriented away from the H_2_O molecule. However, interactions of C3–H3 with the methyl group of the ethyl substituent are present and reflect the proximity of these groups in this conformer. Unsurprisingly, the largest *E* ^(2)^ contribution identified by the NBO analysis (Table S23) is for the primary hydrogen bond interaction, O_w_–H_b_···O1, with a second order stabilization energy of 12.26 kJ mol^–1^. The magnitude of this interaction is ∼1 kJ mol^–1^ greater than identified for the equivalent interaction within the C_1_ conformer. However, the attractive interaction between O_w_ and σ*­(C6–H6) is weaker within the C_s_ conformer (0.25 kJ mol^–1^) than within the C_1_ conformer (0.88 kJ mol^–1^). Overall, the implication is that the aggregated effect of weak interactions within the C_1_ conformer is such that its overall energy is reduced below that of the C_s_ conformer even though the primary (O_w_–H_b_···O1) hydrogen bond is slightly weaker in the C_1_ conformer.

## 4 Discussion

Calculations performed during a previous work^3^ (at the MP2/6-311++G­(d,p) and MP2/6-31G­(d,p) levels of theory), and some performed during the present work (MP2/aug-cc-pVDZ and B3LYP­(D3BJ)/Def2-TZVP levels) predict that the C_1_ conformer of the 2-EF molecule is lower in energy than the C_s_ conformer, but only by a small increment (of the order of 1 kJ mol^–1^ or lower). A further calculation performed during the present work (B3LYP­(D3BJ)/aug-cc-pVTZ) predicts the C_s_ conformer to be the global minimum but finds only a very small difference in energy between the C_s_ and C_1_ conformers (0.1 kJ mol^–1^). Evidently, the difference in energy between these two conformers is small. The nature of a carrier gas influences collisional relaxation within a supersonic expansion as described by Ruoff et al.^54^ Within an expanding gas sample, relaxation from higher-energy to lower-energy conformers occurs through collisional energy transfer and progresses more efficiently when heavier carrier gases are used. The abundances of higher-energy conformers (relative to abundances of lower-energy conformers) are therefore higher where an experiment is performed using helium or neon as a carrier gas rather than argon.^55^ Surveying the collected findings of this and a previous work,^3^ only the C_s_ conformer of 2-EF could be detected when argon was employed as the carrier gas whereas both C_s_ and C_1_ conformers were observed when either neon or helium was used. The experimental results thus imply that the C_s_ conformer of isolated 2-EF is lower in energy than the C_1_ conformer and that the barrier to interconversion between these two conformers is low enough that relaxation to the lower-energy C_s_ geometry proceeds efficiently within an argon expansion.

The relative energies of different conformers can be subtly altered by the effects of weak intermolecular interactions. For example, the minimum-energy geometry of an isolated molecule is sometimes different from that in the hydrated form of the same molecule. This pattern is observed for each of glycidol,^10^ 2-aminoethanol,^11,13^ mevalonolactone,^12^ cysteamine,^14^ formanilide,^15^ sulfanilamide^16^ and 2-hydroxypyridine/2-pyridone.^56,57^ An intramolecular hydrogen bond is present within isolated glycidol^58,59^ but the network of hydrogen bonds changes when a water molecule is added^10^ which leads to a change in the geometry of the glycidol molecule. Within isolated sulfanilamide,^16^ the SO_2_ and NH_2_ groups are eclipsed whereas they are staggered within the observed monohydrate to allow H_2_O to insert between the groups.^16^ The hydration of mevalonolactone prompts reorientation of the hydroxy group and a change in the torsional angle within the molecule’s backbone.^12^ The observations of the present work imply that the geometry of the 2-EF molecule within 2-EF···H_2_O is different from that found within the lowest-energy (C_s_) conformer of the isolated 2-EF molecule. The minimum-energy geometry of 2-EF within 2-EF···H_2_O is C_1_ because of stabilization by weak noncovalent interactions between the H_2_O and 2-EF subunits. This interpretation is supported by the results of the NCI and NBO analyses presented herein.

The structural parameters determined during the present work can be compared with those reported for similar complexes previously. Most complexes formed between furan derivatives and H_2_O contain a hydrogen bond between O1 on furan and an O–H group of H_2_O. Exceptions include microsolvated complexes of furonitrile isomers^60^ where H_2_O molecule(s) interact with carbonyl or nitrile groups rather than with the O atom of the ring. Interesting comparisons can be drawn between the 2-EF···H_2_O complex characterized during the present work and monohydrate complexes identified previously for furfuryl alcohol and furfuryl mercaptan.^6^ 2-EF, furfuryl alcohol and furfuryl mercaptan differ only with respect to the group bound to C6 (CH_3_, OH and SH in 2-EF, furfuryl alcohol and furfuryl mercaptan respectively). The ∠(X–C6–C2–O1) dihedral angle (where X is C, O or S as appropriate to the molecule), which defines the rotation of the substituent relative to the plane of the ring, is similar in each of these molecules.^3,5–7^ A study on furfuryl alcohol···H_2_O and furfuryl mercaptan···H_2_O characterized one conformer of the former and two conformers of the latter. In all of these conformers, H_2_O was found to act as hydrogen bond donor to O1 while simultaneously acting as hydrogen bond acceptor from OH or SH thus forming a bridge between O1 and the OH/SH group. They are therefore similar in shape to the conformer observed herein for 2-EF···H_2_O.

The structural parameters displayed in Table 4 were derived from *r*_0_ coordinates reported for these complexes.^6^ Given the similarities, it is unsurprising that there is only small variation in the values of the *r*(O_W_···O1), *r*(H_b_···O1), ∠(O_W_···O1–C2) and ∠(H_b_···O1–C2). The ∠(O_w_–H_b_···O1) parameter is particularly sensitive to interactions between H_2_O and the group on C2. In all three complexes, the hydrogen bond angle (∠(O_w_–H_b_···O1)) is nonlinear consistent with the presence of hydrogen bonding interaction(s) between H_2_O and the group on C2. The significant difference between ∠(O_w_–H_b_···O1) for furfuryl alcohol···H_2_O and those for the other two complexes featured in Table 4 reflects that H_2_O interacts more strongly with an −OH group than with −CH or −SH groups. The *r*(OH···O_w_) and *r*(H_b_···O1) intermolecular distances were respectively determined to be 1.956(3) and 2.16(1) Å in furfuryl alcohol···H_2_O indicating that the strength of the interaction between the H_2_O and the −OH group is of similar magnitude to that between H_2_O and O1 within that complex. The ∠(O_w_–H_b_···O1) parameter is similar in each of 2-EF···H_2_O and furfuryl mercaptan···H_2_O. Overall, the trend reflects the expected variation in the strength of the C7/O7/S7–H···O_w_ interaction across the series given that O–H groups tend to form stronger hydrogen bonds than S–H and C–H groups.

**Table 4:** Comparison of Experimentally Determined (*r*_0_) Structural Parameters for Complexes Formed Between Furan Derivatives and H_2_O

Parameter	2-EF···H_2_O	furfuryl alcohol···H_2_O	furfuryl mercaptan···H_2_O
*r*(O_W_···O1)/Å	3.0410(34)^a^	2.931(1)^b^	2.926(3)^b^
∠(O_W_···O1–C2) /°	111.50(16)	105.466(19)^c^	110.69(25)^c^
*r*(H_b_···O1)/Å	2.0950(42)	2.155(6)	1.988(24)
∠(H_b_···O1–C2) /°	115.30(11)	117.433(60)	115.47(42)
∠(O_w_–H_b_···O1) /°	167.69(16)	136.060(86)	162.37(79)

a Numbers in parentheses are one standard deviation in units of the last significant figure.

b The parameters listed are those for the GG’-W_da_ isomer of furfuryl alcohol···H_2_O and furfuryl mercaptan···H_2_O (as denoted within ref^6^ which each adopt geometries similar to that of the experimentally observed conformer of 2-EF···H_2_O.

c Results for angles derived using EVAL from *r*_0_ coordinates given in ref 6.

## 5 Conclusions

The broadband microwave spectra of two conformers of the isolated 2-ethylfuran monomer and one isomer of 2-EF···H_2_O have been observed over the frequency range 7.0 – 18.5 GHz. Rotational transitions belonging to the C_s_ conformer of isolated 2-EF were present in spectra acquired using either argon or neon as carrier gases whereas transitions of the C_1_ conformer were present only within the spectrum acquired while using neon. The observations thus suggest that the C_s_ conformer is lower in energy than the C_1_ conformer and that the barrier to interconversion between the two conformers is sufficiently low to allow for relaxation from the C_1_ to the C_s_ form within an argon expansion. The microwave spectra of five isotopologues of 2-EF···H_2_O have been analyzed to determine the molecular geometry of the observed complex. The experimental results are consistent with quantum chemical calculations which find that 2-EF adopts a C_1_ conformation within 2-EF···H_2_O. A change in the relative energy ordering of the conformers of 2-EF thus occurs when the molecule is subsumed into the 2-EF···H_2_O complex. The strongest bond within the monohydrate is the hydrogen bond formed between the O–H group of H_2_O and the oxygen atom of the furan ring. Weaker hydrogen bonds form between the ethyl group on C2 and the O atom of H_2_O and these confer stability upon the C_1_ conformer of the 2-EF molecule within the monohydrate.

## Supplementary Material



## Notes

Tables S1–S15, theoretically calculated spectroscopic parameters and atomic coordinates of optimized geometries of 2-EF and 2-EF···H_2_O; Tables S16–S20, observed and calculated transition frequencies of isotopologues of 2-EF···H_2_O; Table S21, final cycle of fit of structural parameters for 2-EF···H_2_O; Tables S22–23, calculated values and plots of NBO stabilization energy contributions for conformers of 2-EF···H_2_O; Figure S1 Plot of the RDG (a.u.) vs sign­(λ_2_)­ρ, and of NCI isosurfaces for the experimentally observed conformation of 2-EF···H_2_O (PDF)

## #

Department of Chemistry, King’s College London, London, SE1 1DB, U.K
