# Supplementary material for: Hydration and Conformation of 2‑Ethylfuran Explored by Microwave Spectroscopy
Source: J Phys Chem A. 2025 May 14;129(21):4644–53. doi: 10.1021/acs.jpca.5c01281 (PMC12128022; doi:10.1021/acs.jpca.5c01281)
Supplement: Supplementary file 1 [file jp5c01281_si_001.pdf]

*Journal of Physical Chemistry A*

# Supplementary Information

# Hydration and Conformation of 2-Ethylfuran Explored by Microwave Spectroscopy

Charlotte N. Cummings[a] and Nicholas R. Walker*

Chemistry- School of Natural and Environmental Sciences, Newcastle University, Bedson Building, Newcastle-upon-Tyne, NE1 7RU, UK.

KEYWORDS: furan, rotational spectroscopy, microwave spectroscopy, hydrogen bonding, 2-ethylfuran.

AUTHOR INFORMATION:

* Corresponding Author: Nicholas R. Walker, nick.walker@ncl.ac.uk

[a] Present address: Department of Chemistry, King's College London, London, SE1 1DB, U.K

**Table S1** - Spectroscopic parameters of the 2-ethylfuran monomer calculated at different levels of theory.

| | 2-ethylfuran ($C_s$) | |
|---|---|---|
| | $\omega$B97X-D/aug-cc-pVQZ | B3LYP(D3BJ)/aug-cc-pVTZ |
| $A_e$ (MHz) | 7231.9 | 7183.6 |
| $B_e$ (MHz) | 1948.0 | 1931.3 |
| $C_e$ (MHz) | 1564.1 | 1551.0 |
| $\lvert\mu_a\rvert$, $\lvert\mu_b\rvert$, $\lvert\mu_c\rvert$ (D)[b] | 0.55, 0.37, 0.00 | 0.57, 0.44, 0.00 |
| | 2-ethylfuran ($C_1$) | |
| | $\omega$B97X-D/aug-cc-pVQZ | B3LYP(D3BJ)/aug-cc-pVTZ |
| $A_e$ (MHz) | 6800.4 | 6752.5 |
| $B_e$ (MHz) | 1949.9 | 1943.2 |
| $C_e$ (MHz) | 1669.9 | 1656.3 |
| $\lvert\mu_a\rvert$, $\lvert\mu_b\rvert$, $\lvert\mu_c\rvert$ (D)[b] | 0.30, 0.49, 0.04 | 0.27, 0.55, 0.06 |

[b.] Electric dipole moment components along the principal inertial axes.

**Table S2** - Atomic coordinates of the optimised geometry of 2-ethylfuran ($C_s$) calculated at the $\omega$B97X-D/aug-cc-pVQZ level of theory. Hydrogen atom labels indicate the number of the heavy atom to which they are attached (for example, each of the hydrogen atoms attached to C6 is labelled as “H6”).

| | $\omega$B97X-D/aug-cc-pVQZ | | |
|---|---|---|---|
| | *a* /Å | *b* /Å | *c* /Å |
| O(1) | –0.779935 | –1.071752 | –0.000010 |
| C(2) | 0.211567 | –0.147599 | 0.000007 |
| C(3) | –0.322953 | 1.095783 | 0.000008 |
| H(3) | 0.222638 | 2.022883 | 0.000015 |
| C(4) | –1.743893 | 0.926250 | –0.000001 |
| H(4) | –2.496683 | 1.694559 | –0.000002 |
| C(5) | –1.957470 | –0.404734 | –0.000003 |
| H(5) | –2.845926 | –1.009536 | –0.000008 |
| C(6) | 1.604462 | –0.667653 | 0.000017 |
| H(6) | 1.740544 | –1.310693 | 0.872819 |
| H(6) | 1.740537 | –1.310740 | –0.872751 |
| C(7) | 2.649976 | 0.438225 | –0.000016 |
| H(7) | 2.552981 | 1.071242 | 0.881873 |
| H(7) | 3.651968 | 0.012858 | –0.000006 |
| H(7) | 2.552975 | 1.071193 | –0.881939 |

**Table S3** - Atomic coordinates of the optimised geometry of 2-ethylfuran ($C_s$) calculated at the B3LYP(D3BJ)/aug-cc-pVTZ level of theory. Hydrogen atom labels indicate the number of the heavy atom to which they are attached (for example, each of the hydrogen atoms attached to C6 is labelled as "H6").

| | B3LYP(D3BJ)/aug-cc-pVTZ | | |
|---|---|---|---|
| | *a* /Å | *b* /Å | *c* /Å |
| O(1) | –0.783851 | –1.078622 | 0.000015 |
| C(2) | –1.748889 | 0.930344 | –0.000048 |
| C(3) | –0.324765 | 1.099036 | 0.000062 |
| H(3) | 0.217111 | 2.027950 | 0.000104 |
| C(4) | –1.748889 | 0.930344 | –0.000048 |
| H(4) | –2.497961 | 1.702643 | –0.000092 |
| C(5) | –1.971036 | –0.404156 | –0.000004 |
| H(5) | –2.857696 | –1.010609 | –0.000015 |
| C(6) | 1.609775 | –0.667950 | –0.000005 |
| H(6) | 1.746802 | –1.313452 | 0.872482 |
| H(6) | 1.746790 | –1.313471 | –0.872479 |
| C(7) | 2.661192 | 0.437845 | –0.000022 |
| H(7) | 2.565877 | 1.071918 | 0.881727 |
| H(7) | 3.663320 | 0.011239 | –0.000016 |
| H(7) | 2.565875 | 1.071892 | –0.881788 |

**Table S4** - Atomic coordinates of the optimised geometry of 2-ethylfuran ($C_1$) calculated at the $\omega$B97X-D/aug-cc-pVQZ level of theory. Hydrogen atom labels indicate the number of the heavy atom to which they are attached (for example, each of the hydrogen atoms attached to C6 is labelled as "H6").

| | $\omega$B97X-D/aug-cc-pVQZ | | |
|---|---|---|---|
| | *a* /Å | *b* /Å | *c* /Å |
| O(1) | −0.360109 | −0.973991 | −0.234716 |
| C(2) | 0.191718 | 0.264470 | −0.230548 |
| C(3) | −0.754435 | 1.183725 | 0.070249 |
| H(3) | −0.601177 | 2.246033 | 0.146469 |
| C(4) | −1.974912 | 0.463738 | 0.264326 |
| H(4) | −2.941574 | 0.864643 | 0.513515 |
| C(5) | −1.672050 | −0.835412 | 0.066037 |
| H(5) | −2.248376 | −1.742034 | 0.098071 |
| C(6) | 1.645613 | 0.359627 | −0.521630 |
| H(6) | 1.851020 | −0.125374 | −1.478364 |
| H(6) | 1.886886 | 1.415138 | −0.645293 |
| C(7) | 2.524438 | −0.257252 | 0.566850 |
| H(7) | 2.305062 | −1.316630 | 0.690907 |
| H(7) | 3.579016 | −0.156454 | 0.312572 |
| H(7) | 2.356172 | 0.235712 | 1.523884 |

**Table S5** - Atomic coordinates of the optimised geometry of 2-ethylfuran ($C_1$) calculated at the B3LYP(D3BJ)/aug-cc-pVTZ level of theory. Hydrogen atom labels indicate the number of the heavy atom to which they are attached (for example, each of the hydrogen atoms attached to C6 is labelled as "H6").

| | B3LYP(D3BJ)/aug-cc-pVTZ | | |
|---|---|---|---|
| | *a* /Å | *b* /Å | *c* /Å |
| O(1) | –0.344914 | –0.971956 | –0.242066 |
| C(2) | 0.196094 | 0.285865 | –0.222305 |
| C(3) | –0.773062 | 1.187012 | 0.082802 |
| H(3) | –0.639920 | 2.251441 | 0.168610 |
| C(4) | –1.987890 | 0.447595 | 0.262213 |
| H(4) | –2.959805 | 0.836243 | 0.510958 |
| C(5) | –1.671858 | –0.851855 | 0.054307 |
| H(5) | –2.235418 | –1.766210 | 0.074138 |
| C(6) | 1.649901 | 0.396076 | –0.504211 |
| H(6) | 1.861231 | –0.037661 | –1.485611 |
| H(6) | 1.894950 | 1.456543 | –0.569993 |
| C(7) | 2.528493 | –0.284408 | 0.552873 |
| H(7) | 2.293786 | –1.345616 | 0.625202 |
| H(7) | 3.583781 | –0.186017 | 0.297358 |
| H(7) | 2.369854 | 0.163462 | 1.533994 |

**Table S6** - Spectroscopic parameters of 2-EF···$H_2O$ ($C_1$ conformer) calculated at different levels of theory.

| 2-EF···$H_2O$ ($C_1$ conformer) | | | | |
|---|---|---|---|---|
| | *ω*B97X-D/aug-cc-pVQZ | | B3LYP(D3BJ)/aug-cc-pVTZ | |
| $A_e$ (MHz) | 2520.742 | – 0.4 %[a] | 2539.834 | + 0.4 % |
| $B_e$ (MHz) | 1483.172 | + 2.1 % | 1480.461 | + 2.0 % |
| $C_e$ (MHz) | 1027.281 | + 1.7 % | 1016.473 | + 0.6 % |
| $\|\mu_a\|$, $\|\mu_b\|$, $\|\mu_c\|$ (D)[b] | 1.8, 0.1, 0.9 | | 1.8, 0.0, 0.7 | |
| | B3LYP(D3BJ)/Def2-TZVP | | MP2/aug-cc-pVDZ | |
| $A_e$ (MHz) | 2507.921 | – 0.9 % | 2616.067 | + 3.4 % |
| $B_e$ (MHz) | 1482.404 | + 2.1 % | 1442.375 | – 0.7 % |
| $C_e$ (MHz) | 1025.619 | + 1.5 % | 1030.142 | + 1.9 % |
| $\|\mu_a\|$, $\|\mu_b\|$, $\|\mu_c\|$ (D) | 2.0, 0.3, 0.7 | | 1.6, 0.5, 0.3 | |

[a] Calculated by $[(A_e - A_0)/A_0] \times 100$ % where $A_e$ are the results of computational calculations presented above and $A_0$ are the experimental rotational constants.

[b] Electric dipole moment components along the principal inertial axes.

**Table S7** - Spectroscopic parameters of 2-EF···$H_2O$ ($C_s$ conformer) calculated at different levels of theory.

| 2-EF···$H_2O$ ($C_s$ conformer) | | | | |
|---|---|---|---|---|
| | *ω*B97X-D/aug-cc-pVQZ | | B3LYP(D3BJ)/aug-cc-pVTZ | |
| $A_e$ (MHz) | 1926.004 | – 24 %[a] | 1894.604 | – 25 % |
| $B_e$ (MHz) | 1703.675 | + 17 % | 1711.304 | + 18 % |
| $C_e$ (MHz) | 922.305 | – 8.7 % | 923.990 | – 8.5 % |
| $\|\mu_a\|, \|\mu_b\|, \|\mu_c\|$ (D)[b] | 1.7, 0.1, 1.5 | | 1.5, 0.3, 1.5 | |
| | B3LYP(D3BJ)/Def2-TZVP | | MP2/aug-cc-pVDZ | |
| $A_e$ (MHz) | 1879.715 | – 26 % | 1909.812 | – 25 % |
| $B_e$ (MHz) | 1743.009 | + 20 % | 1744.699 | + 20 % |
| $C_e$ (MHz) | 937.908 | – 7.2 % | 968.208 | – 4.2 % |
| $\|\mu_a\|, \|\mu_b\|, \|\mu_c\|$ (D) | 1.7, 0.5, 1.6 | | 1.5, 0.3, 0.1 | |

[a] Calculated by $[(A_e - A_0)/A_0] \times 100$ % where $A_e$ are the results of computational calculations presented above and $A_0$ are the experimental rotational constants.

[b] Electric dipole moment components along the principal inertial axes.

**Table S8** - Atomic coordinates of the optimised geometry of 2-EF···$H_2O$ ($C_1$ conformer) calculated at the $\omega$B97X-D/aug-cc-pVQZ level of theory. Hydrogen atom labels indicate the number of the heavy atom to which they are attached (for example, each of the hydrogen atoms attached to C6 is labelled as "H6").

| | $\omega$B97X-D/aug-cc-pVQZ | | |
|---|---|---|---|
| | *a* /Å | *b* /Å | *c* /Å |
| O(1) | −0.202337 | 0.696740 | −0.213717 |
| C(2) | −0.396520 | −0.562417 | 0.266850 |
| C(3) | −1.722678 | −0.796063 | 0.382752 |
| H(3) | −1.429460 | 2.263243 | −0.784533 |
| C(4) | −2.391869 | 0.392121 | −0.051901 |
| H(4) | −3.451721 | 0.569479 | −0.099772 |
| C(5) | −1.422730 | 1.259698 | −0.400218 |
| H(5) | −2.172797 | −1.706615 | 0.737385 |
| C(6) | 0.818399 | −1.376204 | 0.528373 |
| H(6) | 1.477109 | −0.829388 | 1.206141 |
| H(6) | 0.502239 | −2.281329 | 1.046159 |
| C(7) | 1.589364 | −1.734765 | −0.742991 |
| H(7) | 1.931493 | −0.837135 | −1.255200 |
| H(7) | 2.466749 | −2.331993 | −0.499089 |
| H(7) | 0.962811 | −2.305380 | −1.427955 |
| $H_b$ | 1.637693 | 1.472884 | 0.050482 |
| $O_W$ | 2.564858 | 1.648473 | 0.233421 |
| $H_{nb}$ | 2.564845 | 2.315022 | 0.917698 |

**Table S9** - Atomic coordinates of the optimised geometry of 2-EF···$H_2O$ ($C_1$ conformer) calculated at the B3LYP(D3BJ)/aug-cc-pVTZ level of theory. Hydrogen atom labels indicate the number of the heavy atom to which they are attached (for example, each of the hydrogen atoms attached to C6 is labelled as "H6").

| | B3LYP(D3BJ)/aug-cc-pVTZ | | |
|---|---|---|---|
| | *a* /Å | *b* /Å | *c* /Å |
| O(1) | −0.195267 | 0.717998 | −0.123455 |
| C(2) | −0.409855 | −0.583572 | 0.266068 |
| C(3) | −1.745331 | −0.819374 | 0.293998 |
| H(3) | −1.407727 | 2.321305 | −0.652704 |
| C(4) | −2.398782 | 0.396044 | −0.096849 |
| H(4) | −3.456854 | 0.569828 | −0.184426 |
| C(5) | −1.418546 | 1.294239 | −0.338541 |
| H(5) | −2.212826 | −1.751058 | 0.560765 |
| C(6) | 0.791576 | −1.414825 | 0.530831 |
| H(6) | 1.418510 | −0.918764 | 1.275672 |
| H(6) | 0.449749 | −2.351001 | 0.973266 |
| C(7) | 1.628138 | −1.696196 | −0.724272 |
| H(7) | 2.008862 | −0.770779 | −1.152631 |
| H(7) | 2.483218 | −2.324756 | −0.476154 |
| H(7) | 1.032152 | −2.207842 | −1.480246 |
| $H_b$ | 1.619917 | 1.484939 | 0.091150 |
| $O_W$ | 2.571163 | 1.622233 | 0.196075 |
| $H_{nb}$ | 2.660380 | 2.428093 | 0.711543 |

**Table S10** - Atomic coordinates of the optimised geometry of 2-EF$\cdots$$H_2O$ ($C_1$ conformer) calculated at the B3LYP(D3BJ)/Def2-TZVP of theory. Hydrogen atom labels indicate the number of the heavy atom to which they are attached (for example, each of the hydrogen atoms attached to C6 is labelled as "H6").

| | B3LYP(D3BJ)/Def2-TZVP | | |
|---|---|---|---|
| | *a* /Å | *b* /Å | *c* /Å |
| O(1) | 0.200334 | 0.697577 | 0.212461 |
| C(2) | 0.389886 | −0.576079 | −0.268183 |
| C(3) | 1.721705 | −0.810493 | −0.378364 |
| H(3) | 2.172789 | −1.724671 | −0.725518 |
| C(4) | 2.398026 | 0.378165 | 0.053591 |
| H(4) | 3.460771 | 0.545205 | 0.098458 |
| C(5) | 1.433885 | 1.259598 | 0.400311 |
| H(5) | 1.441696 | 2.265812 | 0.778784 |
| C(6) | −0.826797 | −1.388059 | −0.524603 |
| H(6) | −1.476873 | −0.857254 | −1.226033 |
| H(6) | −0.507532 | −2.308395 | −1.016796 |
| C(7) | −1.623038 | −1.712728 | 0.745211 |
| H(7) | −1.984859 | −0.800471 | 1.218467 |
| H(7) | −2.490252 | −2.328222 | 0.502351 |
| H(7) | −1.005492 | −2.254121 | 1.463719 |
| $H_b$ | −1.611225 | 1.490818 | −0.025913 |
| $O_W$ | −2.536881 | 1.678719 | −0.234494 |
| $H_{nb}$ | −2.514820 | 2.187429 | −1.050785 |

**Table S11** - Atomic coordinates of the optimised geometry of 2-EF···$H_2O$ ($C_1$ conformer) calculated at the MP2/aug-cc-pVDZ of theory. Hydrogen atom labels indicate the number of the heavy atom to which they are attached (for example, each of the hydrogen atoms attached to C6 is labelled as "H6").

| | MP2/aug-cc-pVDZ | | |
|---|---|---|---|
| | *a* /Å | *b* /Å | *c* /Å |
| O(1) | −0.228585 | 0.715221 | −0.243003 |
| C(2) | −0.368712 | −0.561812 | 0.274354 |
| C(3) | −1.712943 | −0.845416 | 0.416030 |
| H(3) | −1.540435 | 2.240705 | −0.826624 |
| C(4) | −2.439291 | 0.313792 | −0.030748 |
| H(4) | −3.517872 | 0.455825 | −0.064550 |
| C(5) | −1.491362 | 1.233869 | −0.420115 |
| H(5) | −2.122601 | −1.778657 | 0.798341 |
| C(6) | 0.890370 | −1.329083 | 0.516251 |
| H(6) | 1.540903 | −0.768807 | 1.208564 |
| H(6) | 0.604311 | −2.268689 | 1.015926 |
| C(7) | 1.655591 | −1.623767 | −0.786409 |
| H(7) | 1.969145 | −0.686695 | −1.269563 |
| H(7) | 2.559907 | −2.215549 | −0.576321 |
| H(7) | 1.021750 | −2.187893 | −1.488604 |
| $H_b$ | 1.581227 | 1.533723 | 0.102100 |
| $O_W$ | 2.529420 | 1.594169 | 0.299425 |
| $H_{nb}$ | 2.660937 | 2.511326 | 0.570066 |

**Table S12** - Atomic coordinates of the optimised geometry of 2-EF$\cdots H_2O$ ($C_s$ conformer) calculated at the *ω*B97X-D/aug-cc-pVQZ level of theory. Hydrogen atom labels indicate the number of the heavy atom to which they are attached (for example, each of the hydrogen atoms attached to C6 is labelled as "H6").

| | *ω*B97X-D/aug-cc-pVQZ | | |
|---|---|---|---|
| | *a* /Å | *b* /Å | *c* /Å |
| O(1) | 0.362762 | 0.835485 | 0.175611 |
| C(2) | −0.440181 | −0.256448 | 0.057435 |
| C(3) | −1.718808 | 0.144057 | −0.118855 |
| H(3) | −2.572975 | −0.497371 | −0.243400 |
| C(4) | −1.706112 | 1.576108 | −0.110720 |
| H(4) | −2.545812 | 2.238457 | −0.225142 |
| C(5) | −0.422239 | 1.938069 | 0.069168 |
| H(5) | 0.080124 | 2.885506 | 0.137521 |
| C(6) | 0.226202 | −1.582260 | 0.142633 |
| H(6) | 1.027083 | −1.622672 | −0.598871 |
| H(6) | 0.716889 | −1.668859 | 1.115237 |
| C(7) | −0.738680 | −2.741769 | −0.058775 |
| H(7) | −1.215507 | −2.689697 | −1.037250 |
| H(7) | −0.208522 | −3.689840 | 0.008227 |
| H(7) | −1.521924 | −2.740646 | 0.699306 |
| $H_b$ | 2.343960 | 0.566464 | −0.042446 |
| $O_W$ | 3.252111 | 0.288964 | −0.191218 |
| $H_{nb}$ | 3.676617 | 0.353835 | 0.662091 |

**Table S13** - Atomic coordinates of the optimised geometry of 2-EF···$H_2O$ ($C_s$ conformer) calculated at the B3LYP(D3BJ)/aug-cc-pVTZ level of theory. Hydrogen atom labels indicate the number of the heavy atom to which they are attached (for example, each of the hydrogen atoms attached to C6 is labelled as "H6").

| | B3LYP(D3BJ)/aug-cc-pVTZ | | |
|---|---|---|---|
| | *a* /Å | *b* /Å | *c* /Å |
| O(1) | 0.526481 | 0.727421 | 0.266411 |
| C(2) | −0.486888 | −0.182229 | 0.078229 |
| C(3) | −1.637166 | 0.486037 | −0.186269 |
| H(3) | −2.597195 | 0.041054 | −0.377164 |
| C(4) | −1.325493 | 1.886689 | −0.163589 |
| H(4) | −2.000247 | 2.707222 | −0.333279 |
| C(5) | −0.006681 | 1.980041 | 0.112424 |
| H(5) | 0.676951 | 2.800466 | 0.226615 |
| C(6) | −0.121613 | −1.617595 | 0.198869 |
| H(6) | 0.703693 | −1.832847 | −0.484682 |
| H(6) | 0.272152 | −1.800882 | 1.203036 |
| C(7) | −1.295255 | −2.552523 | −0.078192 |
| H(7) | −1.680861 | −2.410684 | −1.087925 |
| H(7) | −0.983206 | −3.591208 | 0.019405 |
| H(7) | −2.112693 | −2.379975 | 0.622403 |
| $H_b$ | 2.398227 | 0.123433 | −0.013604 |
| $O_W$ | 3.226310 | −0.312483 | −0.256177 |
| $H_{nb}$ | 3.786793 | −0.246944 | 0.521523 |

**Table S14** - Atomic coordinates of the optimised geometry of 2-EF···$H_2O$ ($C_s$ conformer) calculated at the B3LYP(D3BJ)/Def2-TZVP of theory. Hydrogen atom labels indicate the number of the heavy atom to which they are attached (for example, each of the hydrogen atoms attached to C6 is labelled as "H6").

| | B3LYP(D3BJ)/Def2-TZVP | | |
|---|---|---|---|
| | *a* /Å | *b* /Å | *c* /Å |
| O(1) | −0.278721 | −0.866109 | 0.335161 |
| C(2) | 0.411286 | 0.298308 | 0.098757 |
| C(3) | 1.690334 | −0.005434 | −0.236000 |
| H(3) | 2.466921 | 0.699666 | −0.476349 |
| C(4) | 1.803941 | −1.435651 | −0.208830 |
| H(4) | 2.679916 | −2.023860 | −0.423266 |
| C(5) | 0.587789 | −1.908685 | 0.140292 |
| H(5) | 0.181446 | −2.893255 | 0.285546 |
| C(6) | −0.351499 | 1.564251 | 0.255957 |
| H(6) | −1.251410 | 1.514183 | −0.364285 |
| H(6) | −0.706062 | 1.638614 | 1.289956 |
| C(7) | 0.469226 | 2.800094 | −0.099646 |
| H(7) | 0.803399 | 2.764424 | −1.137670 |
| H(7) | −0.128306 | 3.702371 | 0.030237 |
| H(7) | 1.351532 | 2.886984 | 0.536708 |
| $H_b$ | −2.238405 | −0.794909 | −0.101929 |
| $O_W$ | −3.154717 | −0.565523 | −0.309510 |
| $H_{nb}$ | −3.571173 | −0.405453 | 0.542989 |

**Table S15** - Atomic coordinates of the optimised geometry of 2-EF···$H_2O$ ($C_s$ conformer) calculated at the MP2/aug-cc-pVDZ of theory. Hydrogen atom labels indicate the number of the heavy atom to which they are attached (for example, each of the hydrogen atoms attached to C6 is labelled as "H6").

| | MP2/aug-cc-pVDZ | | |
|---|---|---|---|
| | *a* /Å | *b* /Å | *c* /Å |
| O(1) | −0.307628 | −0.883336 | −0.490782 |
| C(2) | −0.154620 | 0.444062 | −0.127455 |
| C(3) | −1.352490 | 0.918843 | 0.370246 |
| H(3) | −1.881097 | −2.266341 | −0.453931 |
| C(4) | −2.292248 | −0.170448 | 0.314470 |
| H(4) | −3.336665 | −0.168032 | 0.620942 |
| C(5) | −1.608440 | −1.241828 | −0.214374 |
| H(5) | −1.535279 | 1.928585 | 0.731468 |
| C(6) | 1.197266 | 1.046987 | −0.342614 |
| H(6) | 1.948171 | 0.464886 | 0.217263 |
| H(6) | 1.463205 | 0.958728 | −1.410340 |
| C(7) | 1.231963 | 2.517681 | 0.089909 |
| H(7) | 0.994789 | 2.617169 | 1.160696 |
| H(7) | 2.233177 | 2.940948 | −0.078004 |
| H(7) | 0.506555 | 3.116376 | −0.483051 |
| $H_b$ | 1.377880 | −1.906939 | 0.049968 |
| $O_W$ | 2.282794 | −2.048955 | 0.369569 |
| $H_{nb}$ | 2.347267 | −3.003827 | 0.494934 |

**Table S16** - Observed transition frequencies 2-EF···$H_2{}^{16}O$. $\nu_{obs}$ - $\nu_{calc}$ obtained after fitting using Western's PGOPHER program (ref 44).

| $J'$ | $K_{-1}'$ | $K_1'$ | → | $J''$ | $K_{-1}''$ | $K_1''$ | $\nu_{obs}$ (MHz) | $\nu_{obs}$ - $\nu_{calc}$ (MHz) |
|---|---|---|---|---|---|---|---|---|
| 3 | 1 | 3 | → | 2 | 1 | 2 | 6663.597 | -0.013 |
| 3 | 0 | 3 | → | 2 | 0 | 2 | 6985.938 | 0.006 |
| 3 | 2 | 2 | → | 2 | 2 | 1 | 7387.685 | -0.015 |
| 3 | 2 | 1 | → | 2 | 2 | 0 | 7789.448 | 0.003 |
| 3 | 1 | 2 | → | 2 | 1 | 1 | 7969.904 | 0.008 |
| 4 | 1 | 4 | → | 3 | 1 | 3 | 8790.480 | -0.013 |
| 6 | 2 | 5 | → | 6 | 0 | 6 | 8863.474 | -0.005 |
| 4 | 0 | 4 | → | 3 | 0 | 3 | 9010.619 | 0.004 |
| 4 | 2 | 3 | → | 3 | 2 | 2 | 9763.200 | -0.011 |
| 4 | 3 | 2 | → | 3 | 3 | 1 | 10027.175 | -0.001 |
| 4 | 3 | 1 | → | 3 | 3 | 0 | 10129.414 | 0.009 |
| 4 | 1 | 3 | → | 3 | 1 | 2 | 10453.736 | 0.016 |
| 4 | 2 | 2 | → | 3 | 2 | 1 | 10600.389 | 0.011 |
| 5 | 1 | 5 | → | 4 | 1 | 4 | 10872.672 | -0.014 |
| 5 | 0 | 5 | → | 4 | 0 | 4 | 10989.356 | 0.010 |
| 5 | 3 | 3 | → | 4 | 3 | 2 | 12543.093 | 0.000 |
| 5 | 4 | 2 | → | 4 | 4 | 1 | 12556.100 | -0.004 |
| 5 | 4 | 1 | → | 4 | 4 | 0 | 12574.627 | 0.017 |
| 5 | 1 | 4 | → | 4 | 1 | 3 | 12755.746 | 0.007 |
| 5 | 3 | 2 | → | 4 | 3 | 1 | 12866.519 | -0.006 |
| 6 | 1 | 6 | → | 5 | 1 | 5 | 12924.617 | -0.001 |
| 6 | 0 | 6 | → | 5 | 0 | 5 | 12977.491 | 0.005 |
| 5 | 2 | 3 | → | 4 | 2 | 2 | 13373.957 | -0.014 |
| 6 | 2 | 5 | → | 5 | 2 | 4 | 14299.661 | -0.014 |
| 6 | 1 | 5 | → | 5 | 1 | 4 | 14854.491 | 0.005 |
| 7 | 1 | 7 | → | 6 | 1 | 6 | 14959.473 | 0.002 |
| 7 | 0 | 7 | → | 6 | 0 | 6 | 14981.306 | 0.000 |
| 6 | 4 | 3 | → | 5 | 4 | 2 | 15125.780 | 0.001 |
| 6 | 4 | 2 | → | 5 | 4 | 1 | 15204.684 | -0.009 |
| 6 | 2 | 4 | → | 5 | 2 | 3 | 16018.166 | 0.004 |
| 7 | 2 | 6 | → | 6 | 2 | 5 | 16459.065 | 0.012 |
| 8 | 0 | 8 | → | 7 | 0 | 7 | 16994.476 | -0.005 |

**Table S17** - Observed transition frequencies 2-EF⋯$H_2{}^{18}O$. $\nu_{obs}$ - $\nu_{calc}$ obtained after fitting using Western's PGOPHER program (ref 44).

| *J'* | $K_{-1}$' | $K_1$' | → | *J*" | $K_{-1}$" | $K_1$" | $\nu_{obs}$ (MHz) | $\nu_{obs}$ - $\nu_{calc}$ (MHz) |
|---|---|---|---|---|---|---|---|---|
| 3 | 0 | 3 | → | 2 | 0 | 2 | 6739.203 | 0.010 |
| 3 | 2 | 2 | → | 2 | 2 | 1 | 7110.164 | -0.006 |
| 3 | 2 | 1 | → | 2 | 2 | 0 | 7481.133 | -0.001 |
| 3 | 1 | 2 | → | 2 | 1 | 1 | 7668.576 | -0.007 |
| 4 | 1 | 4 | → | 3 | 1 | 3 | 8474.880 | -0.004 |
| 4 | 0 | 4 | → | 3 | 0 | 3 | 8697.843 | 0.004 |
| 4 | 2 | 3 | → | 3 | 2 | 2 | 9400.404 | -0.013 |
| 4 | 3 | 2 | → | 3 | 3 | 1 | 9643.807 | -0.003 |
| 4 | 3 | 1 | → | 3 | 3 | 0 | 9734.212 | 0.005 |
| 4 | 1 | 3 | → | 3 | 1 | 2 | 10067.729 | -0.007 |
| 4 | 2 | 2 | → | 3 | 2 | 1 | 10180.716 | 0.013 |
| 5 | 1 | 5 | → | 4 | 1 | 4 | 10485.357 | -0.009 |
| 5 | 0 | 5 | → | 4 | 0 | 4 | 10607.200 | 0.001 |
| 5 | 3 | 3 | → | 4 | 3 | 2 | 12065.616 | -0.006 |
| 5 | 4 | 2 | → | 4 | 4 | 1 | 12073.601 | 0.005 |
| 5 | 4 | 1 | → | 4 | 4 | 0 | 12089.302 | 0.013 |
| 5 | 1 | 4 | → | 4 | 1 | 3 | 12300.795 | -0.001 |
| 5 | 3 | 2 | → | 4 | 3 | 1 | 12353.981 | 0.009 |
| 6 | 1 | 6 | → | 5 | 1 | 5 | 12466.288 | -0.002 |
| 6 | 0 | 6 | → | 5 | 0 | 5 | 12523.144 | 0.001 |
| 5 | 2 | 3 | → | 4 | 2 | 2 | 12853.786 | -0.003 |
| 6 | 2 | 5 | → | 5 | 2 | 4 | 13782.438 | -0.003 |
| 6 | 1 | 5 | → | 5 | 1 | 4 | 14342.080 | 0.005 |
| 7 | 1 | 7 | → | 6 | 1 | 6 | 14430.049 | 0.005 |
| 7 | 0 | 7 | → | 6 | 0 | 6 | 14454.199 | 0.011 |
| 6 | 4 | 3 | → | 5 | 4 | 2 | 14543.346 | -0.017 |
| 6 | 2 | 4 | → | 5 | 2 | 3 | 15411.957 | 0.002 |
| 7 | 2 | 6 | → | 6 | 2 | 5 | 15870.986 | 0.004 |
| 7 | 1 | 6 | → | 6 | 1 | 5 | 16247.735 | 0.001 |
| 8 | 1 | 8 | → | 7 | 1 | 7 | 16385.149 | 0.001 |
| 8 | 0 | 8 | → | 7 | 0 | 7 | 16394.800 | -0.010 |

**Table S18** - Observed transition frequencies 2-EF···DOH. $\nu_{obs}$ - $\nu_{calc}$ obtained after fitting using Western's PGOPHER program (ref 44).

| $J'$ | $K_{-1}'$ | $K_1'$ | → | $J''$ | $K_{-1}''$ | $K_1''$ | $\nu_{obs}$ (MHz) | $\nu_{obs}$ - $\nu_{calc}$ (MHz) |
|---|---|---|---|---|---|---|---|---|
| 3 | 1 | 3 | → | 2 | 1 | 2 | 6606.747 | -0.013 |
| 3 | 1 | 2 | → | 2 | 1 | 1 | 7915.103 | 0.007 |
| 4 | 1 | 4 | → | 3 | 1 | 3 | 8713.454 | -0.007 |
| 4 | 0 | 4 | → | 3 | 0 | 3 | 8928.383 | 0.002 |
| 4 | 1 | 3 | → | 3 | 1 | 2 | 10376.815 | 0.013 |
| 4 | 2 | 2 | → | 3 | 2 | 1 | 10535.592 | -0.012 |
| 5 | 1 | 5 | → | 4 | 1 | 4 | 10775.381 | -0.009 |
| 5 | 0 | 5 | → | 4 | 0 | 4 | 10887.828 | 0.002 |
| 5 | 2 | 4 | → | 4 | 2 | 3 | 11974.106 | 0.011 |
| 5 | 3 | 3 | → | 4 | 3 | 2 | 12454.522 | -0.013 |
| 5 | 1 | 4 | → | 4 | 1 | 3 | 12653.144 | 0.007 |
| 5 | 3 | 2 | → | 4 | 3 | 1 | 12787.724 | -0.002 |
| 6 | 1 | 6 | → | 5 | 1 | 5 | 12807.327 | 0.000 |
| 6 | 0 | 6 | → | 5 | 0 | 5 | 12857.656 | 0.003 |
| 5 | 2 | 3 | → | 4 | 2 | 2 | 13288.603 | -0.002 |
| 6 | 2 | 5 | → | 5 | 2 | 4 | 14182.770 | 0.012 |
| 6 | 1 | 5 | → | 5 | 1 | 4 | 14725.198 | 0.008 |
| 7 | 1 | 7 | → | 6 | 1 | 6 | 14822.538 | -0.007 |
| 7 | 0 | 7 | → | 6 | 0 | 6 | 14843.100 | 0.013 |
| 6 | 3 | 4 | → | 5 | 3 | 3 | 14910.458 | 0.009 |
| 6 | 3 | 3 | → | 5 | 3 | 2 | 15643.022 | 0.015 |
| 6 | 2 | 4 | → | 5 | 2 | 3 | 15908.180 | -0.022 |
| 7 | 2 | 6 | → | 6 | 2 | 5 | 16320.063 | -0.003 |
| 7 | 1 | 6 | → | 6 | 1 | 5 | 16667.966 | -0.012 |
| 8 | 1 | 8 | → | 7 | 1 | 7 | 16829.705 | 0.007 |
| 8 | 0 | 8 | → | 7 | 0 | 7 | 16837.600 | -0.010 |

**Table S19** - Observed transition frequencies 2-EF···HOD. $\nu_{obs}$ - $\nu_{calc}$ obtained after fitting using Western's PGOPHER program (ref 44).

| $J'$ | $K_{-1}'$ | $K_1'$ | → | $J''$ | $K_{-1}''$ | $K_1''$ | $\nu_{obs}$ (MHz) | $\nu_{obs}$ - $\nu_{calc}$ (MHz) |
|---|---|---|---|---|---|---|---|---|
| 3 | 1 | 2 | → | 2 | 1 | 1 | 7773.219 | 0.012 |
| 4 | 1 | 4 | → | 3 | 1 | 3 | 8566.943 | -0.013 |
| 4 | 0 | 4 | → | 3 | 0 | 3 | 8781.820 | -0.003 |
| 4 | 2 | 3 | → | 3 | 2 | 2 | 9519.235 | -0.008 |
| 4 | 1 | 3 | → | 3 | 1 | 2 | 10194.759 | 0.013 |
| 4 | 2 | 2 | → | 3 | 2 | 1 | 10339.582 | 0.001 |
| 5 | 1 | 5 | → | 4 | 1 | 4 | 10595.602 | -0.005 |
| 5 | 0 | 5 | → | 4 | 0 | 4 | 10709.322 | 0.003 |
| 5 | 2 | 4 | → | 4 | 2 | 3 | 11766.899 | -0.005 |
| 5 | 3 | 3 | → | 4 | 3 | 2 | 12231.351 | 0.005 |
| 5 | 1 | 4 | → | 4 | 1 | 3 | 12437.988 | 0.001 |
| 5 | 3 | 2 | → | 4 | 3 | 1 | 12548.926 | 0.020 |
| 6 | 1 | 6 | → | 5 | 1 | 5 | 12594.675 | -0.005 |
| 6 | 0 | 6 | → | 5 | 0 | 5 | 12646.141 | 0.002 |
| 5 | 2 | 3 | → | 4 | 2 | 2 | 13044.885 | -0.003 |
| 6 | 1 | 5 | → | 5 | 1 | 4 | 14482.158 | 0.010 |
| 7 | 1 | 7 | → | 6 | 1 | 6 | 14577.074 | 0.003 |
| 7 | 0 | 7 | → | 6 | 0 | 6 | 14598.303 | 0.008 |
| 6 | 3 | 4 | → | 5 | 3 | 3 | 14645.425 | -0.015 |
| 6 | 2 | 4 | → | 5 | 2 | 3 | 15623.118 | -0.018 |

**Table S20** - Observed transition frequencies 2-EF···$D_2O$. $\nu_{obs}$ - $\nu_{calc}$ obtained after fitting using Western's PGOPHER program (ref 44).

| *J'* | $K_{-1}$' | $K_1$' | → | *J*" | $K_{-1}$" | $K_1$" | $\nu_{obs}$ (MHz) | $\nu_{obs}$ - $\nu_{calc}$ (MHz) |
|---|---|---|---|---|---|---|---|---|
| 3 | 2 | 1 | → | 2 | 2 | 0 | 7550.137 | -0.007 |
| 3 | 1 | 2 | → | 2 | 1 | 1 | 7720.766 | 0.013 |
| 4 | 1 | 4 | → | 3 | 1 | 3 | 8495.399 | -0.011 |
| 4 | 0 | 4 | → | 3 | 0 | 3 | 8705.964 | -0.002 |
| 4 | 2 | 3 | → | 3 | 2 | 2 | 9449.084 | 0.001 |
| 4 | 1 | 3 | → | 3 | 1 | 2 | 10121.904 | 0.011 |
| 4 | 2 | 2 | → | 3 | 2 | 1 | 10276.251 | 0.007 |
| 5 | 1 | 5 | → | 4 | 1 | 4 | 10505.475 | -0.011 |
| 5 | 0 | 5 | → | 4 | 0 | 4 | 10615.749 | -0.003 |
| 5 | 2 | 4 | → | 4 | 2 | 3 | 11677.477 | 0.001 |
| 5 | 3 | 3 | → | 4 | 3 | 2 | 12146.772 | -0.001 |
| 5 | 1 | 4 | → | 4 | 1 | 3 | 12342.055 | 0.008 |
| 5 | 3 | 2 | → | 4 | 3 | 1 | 12471.831 | 0.011 |
| 6 | 1 | 6 | → | 5 | 1 | 5 | 12486.214 | 0.000 |
| 6 | 0 | 6 | → | 5 | 0 | 5 | 12535.618 | 0.000 |
| 5 | 2 | 3 | → | 4 | 2 | 2 | 12962.107 | -0.001 |
| 6 | 2 | 5 | → | 5 | 2 | 4 | 13831.152 | -0.003 |
| 6 | 1 | 5 | → | 5 | 1 | 4 | 14362.521 | 0.000 |
| 7 | 1 | 7 | → | 6 | 1 | 6 | 14450.578 | 0.009 |
| 7 | 0 | 7 | → | 6 | 0 | 6 | 14470.763 | 0.009 |
| 6 | 3 | 4 | → | 5 | 3 | 3 | 14542.075 | -0.020 |
| 6 | 4 | 3 | → | 5 | 4 | 2 | 14651.122 | -0.005 |
| 6 | 4 | 2 | → | 5 | 4 | 1 | 14731.857 | 0.011 |
| 6 | 2 | 4 | → | 5 | 2 | 3 | 15517.818 | -0.019 |
| 7 | 2 | 6 | → | 6 | 2 | 5 | 15915.094 | 0.009 |
| 7 | 1 | 6 | → | 6 | 1 | 5 | 16256.295 | 0.001 |
| 8 | 1 | 8 | → | 7 | 1 | 7 | 16407.004 | -0.014 |
| 8 | 0 | 8 | → | 7 | 0 | 7 | 16414.806 | 0.007 |

**Table S21** - Results of fits of structural parameters using Kisiel's STRFIT (ref 48).

Input data:

```
=============
18
1   0   0   0  0.0        0.0        0.0       15.9949146
 2   1   0   0  1.3617      0.0        0.0      12.0
 3   2   1   0  1.3516    109.2718     0.0      12.0
 4   3   2   1  1.4313    106.8107   -0.0456    12.0
 5   4   3   2  1.3466    106.0762    0.0214   12.0
 6   3   2   1  1.0759    125.8095  -179.9572   1.0078250
 7   4   3   2  1.0757    127.6675   179.8092    1.0078250
 8   5   4   3  1.0746    133.6035   179.6316    1.0078250
 9   2   3   4  1.4855    133.8431  -178.9807    12.0
10   9   2   3  1.0919    109.1667  -125.7312    1.0078250
11   9   2   3  1.0896    107.5426    -9.7163    1.0078250
12   9   2   3  1.5295    113.2251   112.3647    12.0
13  12   9   2  1.0886    110.8678     60.8718    1.0078250
14  12   9   2  1.089     110.396    -179.8363    1.0078250
15  12   9   2  1.0897    110.8207    -59.6332    1.0078250
16   1   2   3  2.96025   112.16649  162.84678   15.9949146
17  16   1   2  0.96121     8.44858 -169.76096    1.0078250
18  16  17   1  0.95526   104.5      -120.34707   1.0078250
NO OF PARAMETERS  TO BE FITTED:  2
atom no., parameter no.        16 1
atom no., parameter no.        16 2
NO OF CONSTANTS TO BE FITTED:  15
constant,species,value          1 1 2530.5118  H2 16O
constant,species,value          2 1 1451.9822
constant,species,value          3 1 1010.60117
constant,species,value          1 2 2473.8961  H2 18O
constant,species,value          2 2 1395.45246
constant,species,value          3 2 974.62308
constant,species,value          1 3 2502.7183  DOH
constant,species,value          2 3 1443.35048
constant,species,value          3 3 1001.07474
constant,species,value          1 4 2470.1215  HOD
constant,species,value          2 4 1416.6218
constant,species,value          3 4 984.5466
constant,species,value          1 5 2445.5277  D2O
constant,species,value          2 5 1408.16994
constant,species,value          3 5 975.76183

=======================================
after:   4 iterations,   ALAMDA=   0.10E-06

 FINAL RESULTS  OF LEAST  SQUARES  FIT:

         R(16, 1) =     3.041019 +-  0.003473
       A(16, 1, 2) =   111.500951 +-  0.159618
```

Chi-squared = 16.4993820307
Deviation of fit = 1.126580

| Ni | Axis | Iobs | Icalc | Io-c | Bobs | Bcalc | Bo-c |
|---|---|---|---|---|---|---|---|
| 1 | a | 199.71415 | 200.46034 | -0.74619 | 2530.5118 | 2521.0922 | 9.4196 |
| 1 | b | 348.06144 | 348.65271 | -0.59127 | 1451.9822 | 1449.5198 | 2.4624 |
| 1 | c | 500.07760 | 499.31337 | 0.76423 | 1010.6012 | 1012.1480 | -1.5468 |
| 2 | a | 204.28465 | 205.18594 | -0.90129 | 2473.8961 | 2463.0294 | 10.8667 |
| 2 | b | 362.16140 | 363.15961 | -0.99822 | 1395.4525 | 1391.6168 | 3.8357 |
| 2 | c | 518.53790 | 518.34710 | 0.19080 | 974.6231 | 974.9818 | -0.3588 |
| 3 | a | 201.93204 | 202.52595 | -0.59392 | 2502.7183 | 2495.3790 | 7.3393 |
| 3 | b | 350.14296 | 351.68291 | -1.53995 | 1443.3505 | 1437.0303 | 6.3202 |
| 3 | c | 504.83644 | 504.40393 | 0.43252 | 1001.0747 | 1001.9331 | -0.8584 |
| 4 | a | 204.59682 | 206.18696 | -1.59014 | 2470.1215 | 2451.0716 | 19.0499 |
| 4 | b | 356.74942 | 356.79892 | -0.04949 | 1416.6218 | 1416.4253 | 0.1965 |
| 4 | c | 513.31142 | 511.60347 | 1.70795 | 984.5466 | 987.8334 | -3.2868 |
| 5 | a | 206.65438 | 208.00306 | -1.34868 | 2445.5277 | 2429.6710 | 15.8567 |
| 5 | b | 358.89064 | 359.93862 | -1.04798 | 1408.1699 | 1404.0700 | 4.1000 |
| 5 | c | 517.93275 | 516.55899 | 1.37376 | 975.7618 | 978.3568 | -2.5950 |

Correlation coefficients:

| | | 1 | 2 |
|---|---|---|---|
| 1: | R(16, 1) | 1.000 | |
| 2: | A(16, 1, 2) | -0.240 | 1.000 |

---

Final principal coordinates of parent:

| ATOM NO. | A | B | C | MASS |
|---|---|---|---|---|
| 1 | 0.205826 | -0.699540 | -0.220579 | 15.9949146 |
| 2 | 0.416706 | 0.553978 | 0.267736 | 12.0000000 |
| 3 | 1.745674 | 0.766494 | 0.392250 | 12.0000000 |
| 4 | 2.398910 | -0.429641 | -0.044972 | 12.0000000 |
| 5 | 1.418457 | -1.280326 | -0.403273 | 12.0000000 |
| 6 | 2.207797 | 1.668152 | 0.754211 | 1.0078250 |
| 7 | 3.456197 | -0.623118 | -0.087889 | 1.0078250 |
| 8 | 1.411871 | -2.281804 | -0.792841 | 1.0078250 |
| 9 | -0.786978 | 1.385083 | 0.526807 | 12.0000000 |
| 10 | -1.457851 | 0.845000 | 1.197989 | 1.0078250 |
| 11 | -0.459827 | 2.282471 | 1.051111 | 1.0078250 |
| 12 | -1.545142 | 1.762143 | -0.746921 | 12.0000000 |
| 13 | -1.898170 | 0.872627 | -1.265750 | 1.0078250 |
| 14 | -2.414550 | 2.371571 | -0.504748 | 1.0078250 |

| | | | | |
|---|---|---|---|---|
| 15 | -0.905976 | 2.326587 | -1.425387 | 1.0078250 |
| 16 | -2.661596 | -1.604810 | 0.233483 | 15.9949146 |
| 17 | -1.730015 | -1.452727 | 0.051957 | 1.0078250 |
| 18 | -2.666273 | -2.272887 | 0.916251 | 1.0078250 |

Principal coordinates and estimated uncertainties:

| ATOM NO. | A | dA | B | dB | C | dC |
|---|---|---|---|---|---|---|
| 1 | 0.20583 | 0.00177 | -0.69954 | 0.00064 | -0.22058 | 0.00086 |
| 2 | 0.41671 | 0.00123 | 0.55398 | 0.00060 | 0.26774 | 0.00006 |
| 3 | 1.74567 | 0.00167 | 0.76649 | 0.00231 | 0.39225 | 0.00097 |
| 4 | 2.39891 | 0.00118 | -0.42964 | 0.00373 | -0.04497 | 0.00075 |
| 5 | 1.41846 | 0.00296 | -1.28033 | 0.00193 | -0.40327 | 0.00037 |
| 6 | 2.20780 | 0.00358 | 1.66815 | 0.00308 | 0.75421 | 0.00177 |
| 7 | 3.45620 | 0.00154 | -0.62312 | 0.00583 | -0.08789 | 0.00135 |
| 8 | 1.41187 | 0.00512 | -2.28180 | 0.00206 | -0.79284 | 0.00092 |
| 9 | -0.78698 | 0.00292 | 1.38508 | 0.00288 | 0.52681 | 0.00039 |
| 10 | -1.45785 | 0.00217 | 0.84500 | 0.00449 | 1.19799 | 0.00112 |
| 11 | -0.45983 | 0.00494 | 2.28247 | 0.00251 | 1.05111 | 0.00034 |
| 12 | -1.54514 | 0.00314 | 1.76214 | 0.00380 | -0.74692 | 0.00068 |
| 13 | -1.89817 | 0.00152 | 0.87263 | 0.00429 | -1.26575 | 0.00140 |
| 14 | -2.41455 | 0.00440 | 2.37157 | 0.00562 | -0.50475 | 0.00093 |
| 15 | -0.90598 | 0.00396 | 2.32659 | 0.00227 | -1.42539 | 0.00011 |
| 16 | -2.66160 | 0.00426 | -1.60481 | 0.00210 | 0.23348 | 0.00071 |
| 17 | -1.73002 | 0.00410 | -1.45273 | 0.00098 | 0.05196 | 0.00026 |
| 18 | -2.66627 | 0.00496 | -2.27289 | 0.00213 | 0.91625 | 0.00086 |

**Table S22** - NBO stabilisation energy contributions (≥ 0.21 kJ mol$^{-1}$) of 2-EF···$H_2O$ ($C_1$ conformer) calculated at the B3LYP(D3BJ)/aug-cc-pVTZ level of theory.

| Donor NBO | Acceptor NBO | $E^{(2)}$ (kcal mol$^{-1}$) | $E^{(2)}$ (kJ mol$^{-1}$) |
|---|---|---|---|
| σ(C4-C5) | σ*($H_b$-$O_w$) | 0.05 | 0.21 |
| σ(C2-C3) | σ*($H_b$-$O_w$) | 0.06 | 0.25 |
| σ(C7-H7) | σ*($O_w$-$H_{nb}$) | 0.05 | 0.21 |
| **LP(1) O1** | **σ*($H_b$-$O_w$)** | **2.85** | **11.92** |
| **LP(2) O1** | **σ*($H_b$-$O_w$)** | **0.23** | **0.96** |
| σ($H_b$-$O_w$) | σ*(O1-C5) | 0.08 | 0.33 |
| LP(1) $O_W$ | σ*(O1-C5) | 0.05 | 0.21 |
| **LP(2) $O_w$** | **σ*(C6-H6)** | **0.21** | **0.88** |
| **LP(2) $O_w$** | **σ*(C6-H6)** | **0.08** | **0.33** |

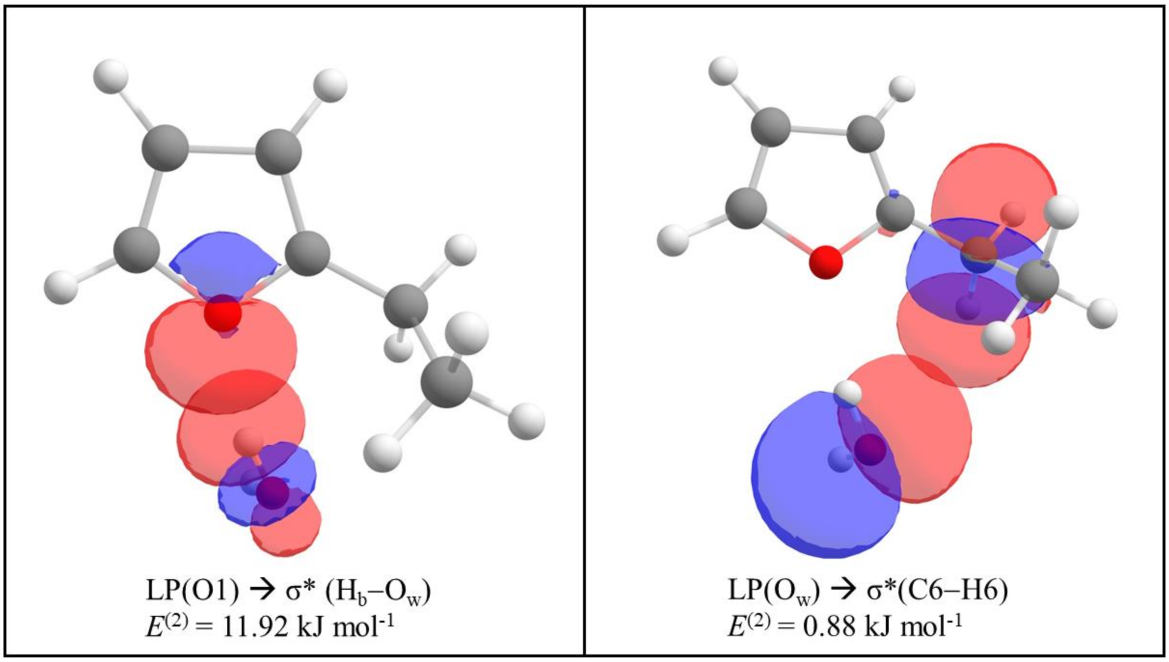

LP(O1) → σ* ($H_b$–$O_w$)
$E^{(2)}$ = 11.92 kJ mol$^{-1}$

LP($O_w$) → σ*(C6–H6)
$E^{(2)}$ = 0.88 kJ mol$^{-1}$

**Table S23** - NBO stabilisation energy contributions (≥ 0.21 kJ mol$^{-1}$) of 2-EF···$H_2O$ ($C_s$ conformer) calculated at the B3LYP(D3BJ)/aug-cc-pVTZ level of theory.

| Donor NBO | Acceptor NBO | $E^{(2)}$ (kcal mol$^{-1}$) | $E^{(2)}$ (kJ mol$^{-1}$) |
|---|---|---|---|
| σ(C4-C5) | σ*($H_b$-$O_w$) | 0.05 | 0.21 |
| σ(C2-C3) | σ*($H_b$-$O_w$) | 0.06 | 0.25 |
| **LP(1) O1** | **σ*($H_b$-$O_w$)** | **2.93** | **12.26** |
| **LP(2) O1** | **σ*($H_b$-$O_w$)** | **0.16** | **0.67** |
| σ($H_b$-$O_w$) | σ*(O1-C5) | 0.08 | **0.33** |
| LP(1) $O_w$ | σ*(O1-C5) | 0.05 | 0.21 |
| **LP(2) $O_w$** | **σ*(C6-H6)** | **0.06** | **0.25** |
| LP(2) $O_w$ | σ*(C6-C7) | 0.10 | 0.42 |

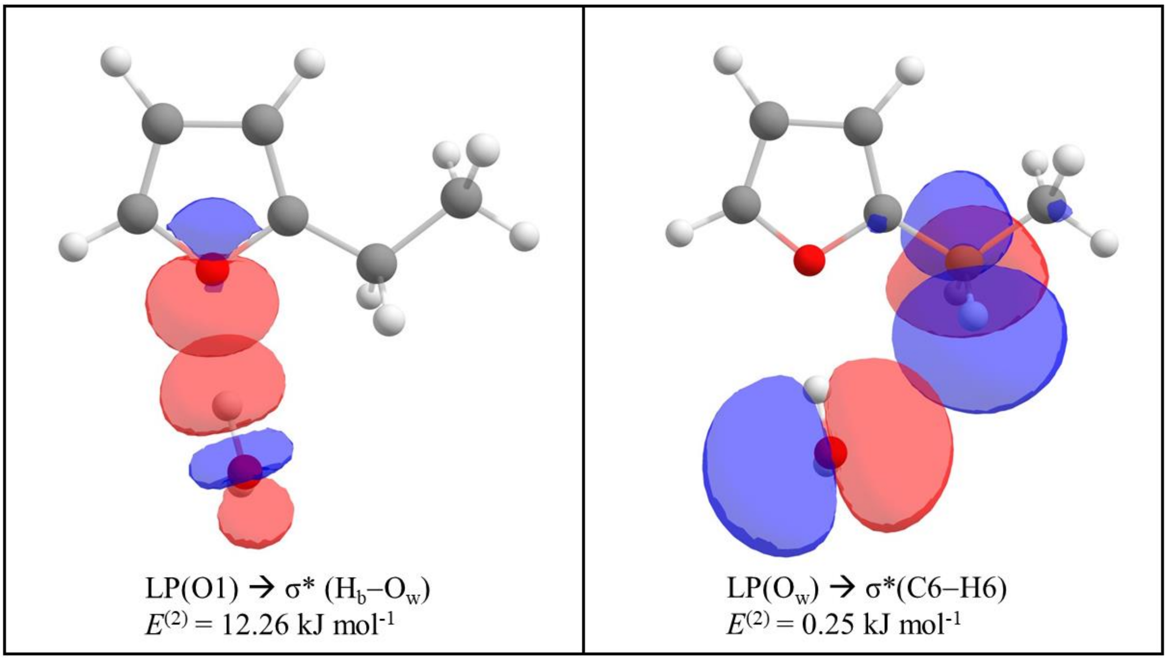

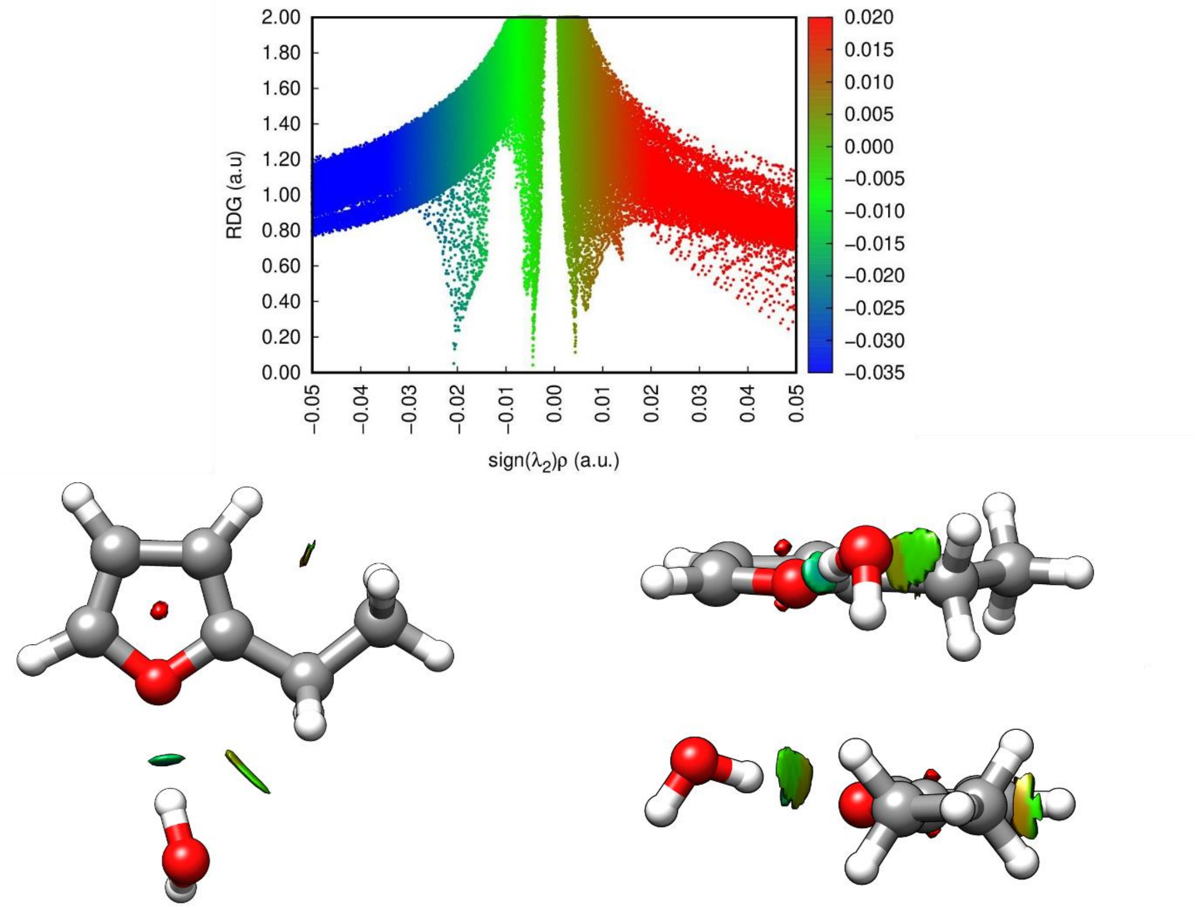


**Figure S1** - Plot of the RDG (a.u.) vs sign($\lambda_2$)$\rho$ (top) and the NCI isosurfaces (bottom) of the $C_s$ conformer of 2-EF···$H_2O$ (which was not observed experimentally). Positive and negative values of sign($\lambda_2$)$\rho$ respectively denote repulsive (red) and attractive (blue) interactions. The isosurface *s* value is 0.5 au.
